# The effectiveness of intravenous (IV) to oral (PO) antibiotic switch (IVOS) interventions in managing community- and hospital-acquired pneumonia—a systematic review

**DOI:** 10.1093/jacamr/dlag065

**Published:** 2026-05-12

**Authors:** Nketia Obed-Arthur, Rosemary H M Lim, Louise Dunsmure

**Affiliations:** School of Pharmacy, University of Reading, Whiteknights Campus, Reading, England RG6 2AH, UK; School of Pharmacy, University of Reading, Whiteknights Campus, Reading, England RG6 2AH, UK; School of Pharmacy, University of Reading, Whiteknights Campus, Reading, England RG6 2AH, UK; Oxford University Hospitals NHS Foundation Trust, John Radcliffe Hospital, Headley Way, Headington, Oxford OX3 9DU, UK

## Abstract

**Background:**

Prolonged intravenous (IV) antibiotic therapy in hospitalized patients with pneumonia increases healthcare costs and hospital length of stay compared to oral (PO) therapy. Antimicrobial stewardship (AMS) interventions promoting timely IV-to-PO antibiotic conversion may reduce healthcare burden without compromising patient outcomes. Optimal design and implementation of such interventions in different healthcare settings, including respiratory medicine remain unclear.

**Objectives:**

The aim of this study was to evaluate the effectiveness of interventions designed to improve IV-to-PO antibiotic switching practices in managing community-acquired pneumonia (CAP) and hospital-acquired pneumonia (HAP).

**Methods:**

The review was registered with PROSPERO (CRD420251039180). PubMed, Scopus, Web of Science, The Cochrane Library databases were searched for studies published from 1995 to 2024 focusing on CAP and/or HAP management or antimicrobials indicated for respiratory infections. Two reviewers independently screened studies using *a priori* inclusion/exclusion criteria. Study quality was assessed using the Mixed Methods Appraisal Tool. Data were analysed using narrative synthesis. Clinical outcomes (cure rates, mortality, length of stay), economic outcomes (cost savings), and process measures (switching rates, IV antibiotic duration) were examined.

**Results:**

58 studies were included. Interventions included clinical guideline/protocol implementation (*n = *40), pharmacist-led interventions (*n = *21), educational programmes (*n = *7), computerized decision support systems (*n = *5), audit/feedback systems (*n = *3), and multidisciplinary team approaches (*n = *1). Most studies demonstrated 1–4-day reductions in IV antibiotic duration, 1–2 day reductions in hospital length of stay, and cost savings, while maintaining equivalent clinical outcomes. Multimodal approaches outperformed single interventions.

**Conclusion:**

Existing AMS interventions effectively promote IV-to-PO switching in CAP/HAP management. Healthcare systems should consider more than one intervention to optimize IV-to-PO antibiotic switch.

## Introduction

Antimicrobial stewardship (AMS) refers to a coordinated set of strategies that promote the optimal use of antimicrobial agents to improve patient outcomes, curb antimicrobial resistance (AMR), and minimize the spread of Multi-Drug Resistance (MDR) infections.^1,2^ One of the aims of AMS is to ensure that patients receive the ‘right’ antimicrobial at the correct dose, via the most appropriate route, for an optimal duration of time. This approach avoids unnecessary exposure to antimicrobials, and reduces the risk of complications such as catheter-related bloodstream infections or prolonged hospitalization.^1,2^ Among various AMS interventions, intravenous (IV) to oral (PO) antibiotic switching (IVOS) is a practical strategy. The conversion of intravenous to oral antibiotics not only reduces healthcare costs and length of stay, but also mitigates risks inherent to prolonged IV therapy such as phlebitis, decreased patient mobility and increased nursing workload.^3,4^ IV medications have a substantially larger environmental footprint compared to oral formulations, with greater carbon emissions associated with manufacturing, cold-chain storage requirements, plastic waste from administration sets and infusion bags, and increased energy consumption for preparation, delivery and subsequent disposal.^5^ The traditional approach to treating patients hospitalized due to infection often relied on IV therapies; this perception was developed in part as early oral antibiotics had limited efficacy due to their poor bioavailability and high acid-lability, resulting in intolerable side effects.^6^ Between the 1950s, when this belief was more widespread and 2025, the availability of highly bioavailable oral antibiotics with comparable efficacy to their IV counterparts has created opportunities for earlier transition to oral therapy. The significance of IV-to-oral switching (IVOS) has been acknowledged at the national policy level in the United Kingdom, serving as a Commissioning for Quality and Innovation (CQUIN) indicator for two years^7^ and supported by consensus criteria for appropriate switching.^8^ Despite established guidance and demonstrated clinical equivalence between intravenous and several oral formulations of antibiotic therapy, IVOS implementation remains inconsistent across healthcare settings, indicating that this continues to be a current challenge requiring further work to achieve consistent, evidence-based practice.^9^

### Pneumonia as a model for IV to PO switch (IVOS) therapy

Community-acquired pneumonia (CAP) and hospital-acquired pneumonia (HAP) serve as effective models for studying IV-to-PO antibiotic switch interventions due to their high prevalence and the significant burden they place on healthcare systems worldwide.^10,11^ Pneumonia is one of the leading causes of hospital admission worldwide^12^; in the United Kingdom alone, CAP accounts for approximately 29 000 deaths annually.^13^ The management of CAP and HAP typically involves empirical antibiotic therapy, with prescribing choices guided by local resistance patterns and severity assessment tools such as CURB-65 and the Pneumonia Severity Index (PSI).^14^ Empirical treatment of moderate to severe pneumonia often starts with IV antibiotics because of the initial severity of the illness and the need for rapid therapeutic concentrations.^15^ However, once patients achieve clinical stability—a physiological state often determined by assessing symptom resolution, an early switch to oral antibiotics becomes feasible.^10,11^ The evidence base demonstrates that early conversion from IV to PO therapy not only maintains clinical efficacy, but also contributes to more efficient resource utilization and reduced healthcare costs.^3^ This predictable clinical trajectory underscores the rationale for more widespread use of IV-to-PO switch interventions as a key component of both AMS and pneumonia management.

### Barriers to timely switching

Despite evidence advocating for the use of IV-to-PO antibiotic switches, studies have also shown that less than half of patients who are eligible candidates for oral antibiotic therapy are actively switched from IV-to-PO.^16,17^ Current barriers to more widespread facilitation of IV-to-PO switches are clinician attitudes that favour IV therapy, unfamiliarity with AMS guidelines and a lack of anticipated positive outcomes.^18,19^ Some clinicians perceive IV medications as having ‘mythical properties’, with beliefs that they achieve superior efficacy and faster clinical cure than oral equivalents, despite pharmacokinetic data suggesting otherwise.^20^ Respiratory clinicians often modify traditional antimicrobial practices when managing pleural infections due to concerns regarding the adequacy of oral antibiotic penetration into pleural tissue. Niwa and colleagues examined the penetration of carbapenems in chemically induced pleurisy and demonstrated that the degree of pleural penetration is influenced by the stage and severity of inflammation.^21^ Similar observations have been made in studies of pleural empyema, where limited data on the efficacy of oral regimens in achieving therapeutic concentrations in the pleural space have resulted in a clinical preference for intravenous (IV) administration.^22^ This concern, although not always supported by direct comparative data of oral versus IV pharmacokinetics in the pleural environment, may have contributed to a departure from more traditional practices that might otherwise favour oral therapy once bioequivalence is assumed. Understanding and addressing these barriers is essential for enhancing AMS and improving patient outcomes.^23^ Conversely, given the established efficacy of oral antibiotics in treating pneumonia and the persistent gap between evidence and practice, there is a critical need to systematically evaluate existing IV-to-PO switch interventions and understand the factors that influence their implementation.

This systematic review aims to assess whether IV-to-PO antibiotic interventions are effective in managing patients in hospital who are being treated for CAP or HAP. The review also aims to understand the developmental process and rationale behind these interventions. By examining what barriers or challenges intervention developers identified and designed their interventions to overcome, we can gain insight into the underlying reasons why earlier IV-to-PO antibiotic switches are not occurring in routine clinical practice. This may also inform future work with regards to either developing new interventions or optimizing existing ones. Understanding which implementation strategies are deemed ‘most effective’, and identifying barriers to sustained adoption of interventions, and quantifying the impact on key clinical and economic outcomes are essential for optimizing antibiotic stewardship efforts globally.

## Methods

### Search strategy

The protocol was registered with PROSPERO (CRD420251039180). The Preferred Reporting Items for Systematic Reviews and Meta-Analyses (PRISMA) was used to report this review (Table S1, available as Supplementary data at *JAC-AMR* Online). A comprehensive systematic literature search was conducted across four major electronic databases: PubMed (including MEDLINE), Scopus, Web of Science, and The Cochrane Library (including the Cochrane Database of Systematic Reviews and Cochrane Central Register of Controlled Trials). The search encompassed studies published between January 1995 and December 2024, with the starting date selected to capture the period following the development of highly bioavailable oral antibiotics and the emergence of formal antimicrobial stewardship programmes.^24^ The complete search strategy is detailed in Table S2 (available as supplementary data). The search was limited to studies published in English due to resource constraints for translation.

### Study selection

Studies were included provided that the population consisted of hospitalized patients (adult and/or paediatric) with CAP, HAP, or the study included the use of antibiotic agents that could be used to manage respiratory infection. Studies must also have examined interventions aimed at facilitating or evaluating IV-to-PO antibiotic switches. Eligible comparators included usual care, historical controls, or alternative implementation strategies. Studies must have reported at least one relevant outcome: IV antibiotic duration, total antibiotic duration, length of stay, clinical cure rates, mortality, readmission rates, adverse events, healthcare costs, or process measures (switching rates, time to switch, guideline adherence). Studies were excluded if they examined only antibiotic selection or dosing without addressing the route of administration, were conference abstracts or opinion pieces without original data, were case reports, or lacked sufficient data for outcome extraction.

### Screening of studies

All search results were imported into Rayyan, a web-based screening tool for systematic reviews. The primary reviewer (NOA) screened all titles and abstracts against predefined eligibility criteria, with duplicates removed. A secondary reviewer (RL/LD) independently screened a random 10% sample, with reviewers blinded to each other’s decisions. Discrepancies were resolved through consensus discussion with a third reviewer via Microsoft Teams. Studies deemed eligible after title/abstract screening advanced to full-text review. The primary reviewer assessed all full-text articles using the inclusion/exclusion criteria. Two reviewers independently assessed 10% of full texts, with discrepancies resolved through discussions to reach consensus.

### Quality assessment

Methodological quality and risk of bias were independently assessed by two reviewers using the Mixed Methods Appraisal Tool (MMAT) version 2018, chosen for its suitability for use across diverse study designs. None of the studies were excluded based on MMAT results. Quality assessment informed the narrative synthesis, with studies exhibiting significant methodological concerns interpreted with caution and their limitations explicitly discussed. Disagreements were resolved through discussion or consultation with a third reviewer. The results of the MMAT can be found in Table S3 (Table S3).

Data extractionData extraction was performed on Rayyan, with data then exported into a standardized data extraction form that was developed in Microsoft Excel. One reviewer (NOA) independently extracted data from all included studies, including: study characteristics (author, year, country, setting, sample size); population characteristics (patient demographics, infection type); intervention details (type, components, personnel involved, switch criteria, timing, duration); and outcome data (clinical outcomes such as mortality and readmissions, process outcomes including switching rates and guideline adherence, length of stay, IV therapy duration, total antibiotic duration, economic outcomes).

### Data synthesis

Due to heterogeneity in study design, interventions, populations, outcome definitions, and measurement methods, a narrative synthesis was employed.^25^ The synthesis was structured around key areas: clinical and economic outcomes, safety of IV-to-PO switch therapy, effectiveness of implementation strategies, and barriers to implementation. Within each area, studies were grouped by intervention type, patient population, or healthcare setting to facilitate comparison and pattern identification.

## Results

**Figure 1.**
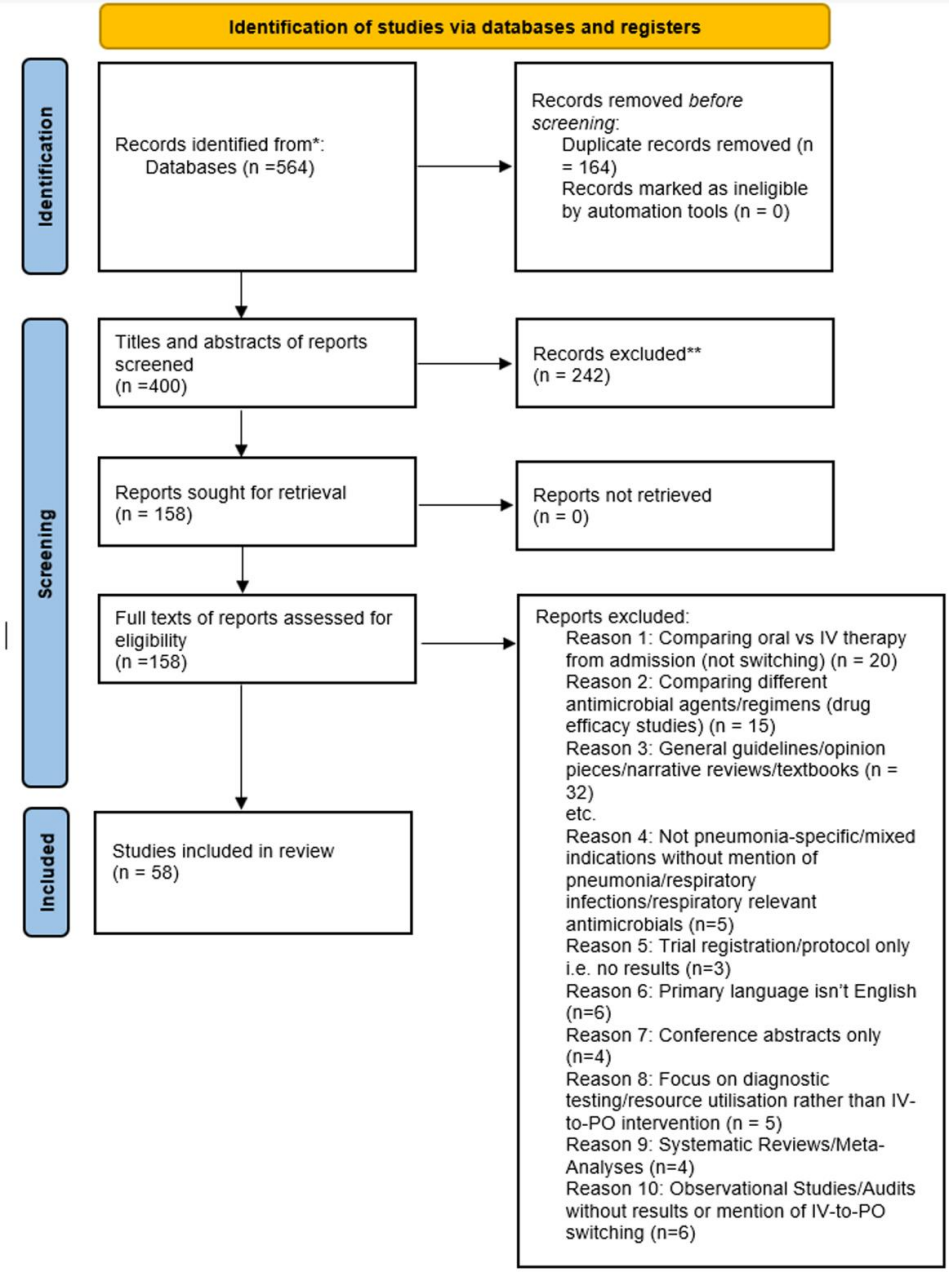
Study selection process.

The study selection process is presented in Figure 1. The initial systematic literature search yielded 400 results. Following removal of duplicates and initial title/abstract screening, 158 full-text articles were assessed for eligibility. Of these, 58 studies met the inclusion criteria and were included in the final narrative synthesis.

### Characteristics of included studies

The characteristics of included studies are detailed in Table 1. The studies included were conducted between 1995 and 2024. This frame captures studies conducted between the first IV-to-oral switch guidelines were introduced^81^ and 2024 when the UK Health Security Agency developed the first national consensus criteria for IV-to-oral switch.^7^ The included studies spanned 27 countries. Most of the studies were conducted in the United States of America (*n = *17). Of the 58 studies included, 51 (87.9%) were conducted in adult populations (defined as individuals aged 18 years and above), three (5.2%) in paediatric populations (aged under 18 years), and four (6.9%) in mixed populations comprising both adults and children. Studies were predominantly conducted in tertiary care hospitals (*n = *28, 48%) and teaching hospitals (*n = *22, 38%). Most studies that implemented or evaluated the efficacy of an AMS intervention (*n = *48, 83%) were conducted in general medical wards, with additional studies in surgical wards (*n = *8, 14%), intensive care units (*n = *7, 12%), and paediatric wards (*n = *6, 10%). The primary infection types studied were community-acquired pneumonia (*n = *44, 76%), hospital-acquired pneumonia (*n = *8, 14%), and mixed respiratory tract infections (*n = *6, 10%). The 58 included studies reported diverse interventions to promote IV to PO antibiotic switching. Interventions were categorized as: clinical guideline/protocol implementation (*n = *40), pharmacist-led interventions (*n = *21), educational programmes (*n = *7), computerized decision support systems (*n = *5), audit/feedback systems (*n = *3), and multidisciplinary team approaches (*n = *1). Intervention types were not mutually exclusive, with some studies implementing multiple interventions (*n = *16). The most common combination was ‘Pharmacist-Led’ and ‘Clinical Guidelines/Protocol Implementation.’ (*n = *6).

**Table 1.** Characteristics and findings of included studies

Authors	Year	Country	Intervention type	Intervention developed	Infection type	Outcomes
Adult Populations						
Grill, E. *et al*.^26^	2011	Germany	Pharmacist-Led Intervention	A ward pharmacist judged whether drug utilization was appropriate within 24 hours after initiating antimicrobial therapy.	Pneumonia (CAP/HAP)	Reduction in the length of antimicrobial courses overall (10 days versus 11 days). Reduction in IV antibiotic duration (8 days versus 10 days). Reduced costs for organization due to decreased cost of antimicrobials (16% decrease).
Oosterheert *et al*.^27^	2006	The Netherlands	Clinical Guidelines and Protocol Implementation	A computer randomized patients to either receive an IV-to-PO switch of antibiotics or receive the standard regimen of intravenous antibiotic therapy.	CAP	Reduction in IV antibiotic duration by 3.4 days. Reduction in the length of hospital stay by 1.9 days. Comparative cure rates of 83%/85%.
Buyle, F.M. *et al*.^28^	2012	Germany, Austria, Belgium	Pharmacist-Led Intervention/Clinical Guidelines and Protocol Implementation	Appropriateness of intravenous administration of antibiotics was reviewed by a physician or pharmacist trained in antimicrobial stewardship against pre-defined criteria.	Respiratory-relevant antimicrobials/Mixed Infections, inclusive of respiratory tract infections	37% of cases of intravenous prescribing on average were deemed inappropriate, suggesting a potential decrease in intravenous prescribing by this amount. This quality indicator was deemed feasible across three European countries (Germany, Austria and Belgium).
van Niekerk, A.C. *et al*.^29^	2011	South Africa	Pharmacist-Led Intervention/Clinical Guidelines and Protocol Implementation	A guideline was developed and integrated into ward rounds by a ward pharmacist over a 7-week period. A pre-intervention and post-intervention approach was taken.	Mixed Infections inclusive of respiratory tract infections	Reduced IV therapy duration (7.2 days to 5.2 days). Shorter length of stay in hospital (11.5 days versus 10.7 days). Cost savings demonstrated (£1414.41).
Emerson, C.R. *et al*.^30^	2008	United States of America	Clinical Guidelines and Protocol Implementation	Analysis of antimicrobial therapy for 100 patients admitted to hospital for pneumonia treatment was compared to analysis for a similar group in which clinical guidelines have been implemented.	Pneumonia (CAP/HAP)	An estimated cost saving of nearly $300 000 annually because of the implementation of the treatment guidelines for pneumonia. A decrease in length of stay by 1 day.
Yaqub, A and Khan, Z^31^	2005	Pakistan	Clinical Guidelines and Protocol Implementation	A computer randomized patients to either receive an IV-to-PO switch of antibiotics or receive the standard regimen of intravenous antibiotic therapy	CAP	Mean duration of treatment was equal for both groups (8 days). Mean length of stay was significantly shorter in those switched to oral antibiotics (4.1 days versus 8.2 days).
Castro-Guardiola, A *et al*.^32^	2001	Spain	Clinical Guidelines and Protocol Implementation	Randomized patients to either receive an IV-to-PO switch of antibiotics once they met specific criteria (afebrile) or receive oral antibiotics.	CAP	Fewer adverse events in the oral and early-switch groups (16% versus 34%). Cost savings mainly due to shorter length of stay (6 days versus 11 days).
Van Der Eerden, MM *et al*.^33^	2004	The Netherlands	Clinical Guidelines and Protocol Implementation	Microbiological and serological tests were performed, with symptoms of CAP, CRP and WCC ordered also. When the patient’s temperature had been normalized for 72 hours and respiratory symptoms had improved, they go.	CAP	Intervention group switch rate 97%. Clinical cure rates maintained (∼97%).
Hendrickson, J.R. *et al*.^34^	1995	United States of America	Pharmacist-Led Intervention/Clinical Guidelines and Protocol Implementation	A clinical pharmacist would review patients to be considered for step-down therapy from IV-to-PO provided they met specific criteria.	CAP	Reduction in total duration of antimicrobial therapy (9.1 days versus 11.9 days). Cost saving of $46.05 per patient achieved.
Xu S *et al*.^35^	2021	China	Computerized Decision Support System/Pharmacist-Led Interventions	This was an observational pre and post-intervention study. In the first phase, pharmacists implemented the conventional practice of reviewing charts and verbally making recommendations to prescribers of IV to PO switches. In the second phase, pharmacists implemented a new intervention practice to inform prescribers via electronic computerized reminders of IV-to-PO switches.	CAP	Length of IV antibiotic therapy decreased more with (12.05 days versus 10.75 days). electronic reminders rather verbal reminders. Length of hospital stay was significantly shorter using electronic reminders. (6.02 days versus 7.40 days).
Frei CR *et al*.^36^	2006	United States of America	Clinical Guidelines and Protocol Implementation	Patients were stratified into guideline-concordant groups and discordant groups based on the American Thoracic Society (ATS) guidelines.	CAP	Guideline concordance was associated with a significant decrease in time to switch from IV-to-PO antibiotics (4.5 days versus 5.9 days), length of stay (5.0 days versus 6.2 days) and in-hospital mortality (3% versus 7%). Guideline concordance was associated with a decrease in time to clinical stability (4% to 1%).
Mazzola, J.L. *et al*.^37^	2005	United States of America	Clinical Guidelines and Protocol Implementation	A retrospective chart review was performed to evaluate the conversion from intravenous to oral antibiotics in the context of the CAP guideline.	CAP	Of the patients reviewed, 66% (*n = *109) received guideline-recommended initial antibiotic(s), route, and dose.
Shrayteh, ZM *et al*.^17^	2014	Lebanon	Clinical Guidelines and Protocol Implementation	This was a retrospective observational study conducted across three hospitals. Practice was evaluated against pre-defined eligibility criteria.	Respiratory-relevant antimicrobials/Mixed Infections inclusive of respiratory tract infections	Among 452 intravenous antibiotic courses from 356 patients who were eligible for conversion, only one third were switched and the others continued intravenous antibiotics beyond day 3.
van den Bergh D *et al*.^38^	2020	South Africa	Pharmacist-Led Interventions/Education	Guidelines were used to create a CAP bundle that pharmacists followed to audit compliance. Training workshops were delivered by AMS-trained pharmacists, and would give feedback to physicians treating patients for CAP.	CAP	Post-implementation, CAP bundle compliance improved (47.8% to 53.6%), which was associated with an improvement in antibiotic choice and duration. There was no difference in mortality.
Fok MC *et al*.^39^	2002	Canada	Clinical Guidelines and Protocol Implementation	A retrospective chart review of patients was conducted.	CAP	53% of empirical choices were consistent with the Canadian guidelines. An additional 24% of patients could have undergone step down, which could have equated to a cost saving of $220 000.
Buyle, F. *et al*.^40^	2010	Belgium	Clinical Guidelines and Protocol Implementation/Education/Pharmacist-Led Intervention	Intervention 1: Guidelines were publicized to all prescribers, and a pre- and post-intervention measurement of fluoroquinolone prescribing was conducted. Intervention 2: An educational interactive session was conducted by infectious disease specialists to medical staff. Intervention 3: Pharmacists attached pre-printed notes with IV-to-PO switch interventions on the front of patients’ charts when they met the criteria.	Respiratory-relevant antimicrobials/Mixed infections that contain respiratory infection	In intervention 1, patients were treated by IV antibiotics 4.1 days longer than necessary, followed by 3.5 days and 1 day longer than necessary in interventions 2 and 3, respectively. The mean additional cost for additional intravenous treatment decreased from €188.0 to €103.0, to €44 in intervention groups 1,2, and 3, respectively.
Hagaman, JT *et al*.^41^	2005	United States of America	Clinical Guidelines and Protocol Implementation	Physician awareness and usage of the American Thoracic Society Guidelines was surveyed, and the guidelines subsequently implemented. Chart reviews were then conducted to assess compliance.	CAP	Physician awareness increased use of guidelines from 5% to 40%. Switch therapy increased from 60% to 86%. Decrease in length of stay by 1.2 days.
Lee RW *et al*.^42^	2006	Australia	Clinical Guidelines and Protocol Implementation	Guidelines were implemented one month prior to evaluating compliance with guidelines.	CAP	Length of stay was significantly shorter in the guideline-concordant group compared to baseline (7.62 days versus 8.36 days). Duration of IV antibiotics was also shorter in the guideline-concordant group compared to baseline. Patient satisfaction was 93.9% in those switched.
Mouwen, AMA *et al*.^16^	2020	The Netherlands	Clinical Guidelines and Protocol Implementation/Education/Computerized Decision Support System	Physicians were educated on the guidelines to use to guide IV-to-PO switches, supported by pocket-sized cards. Physicians were also notified electronically when a switch from IV-to-PO switches should be considered on the patient’s electronic system.	Respiratory-relevant antimicrobials/Mixed Infections	Duration of IV antibiotics was shorter by 2 days following IV-to-PO switches. Length of stay decreased by 1 day.
Lesprit, P. *et al*.^43^	2010	France	Education/Computerized Decision Support System	Prescriptions were screened from a computer-generated listing that prompted physicians to review antibiotic prescriptions. Different strategies involved sending a prompt, and sending a prompt coupled with infectious disease physician advice.	CAP/HAP/Mixed Infections inclusive of respiratory tract infections	More prescriptions were modified in cases where infectious disease physician advice followed an electronic prompt, than when an electronic prompt was issued on its own.
Ahmed SA *et al*.^44^	2018	India	Education/Clinical Guidelines and Protocol Implementation	A before-and-after approach was taken. Interventions included education and introduction of an antibiogram.	VAP (type of HAP)	There was an improvement in the number of patients who underwent de-escalation of antibiotics (21% versus 36%), appropriate antibiotic use (25% versus 46%), IV-to-PO switch and decrease in expenditure (₹24 705.5 versus ₹16 517.5)
Dunn, K *et al*.^4^	2011	Ireland	Clinical Guidelines and Protocol Implementation/Pharmacist-Led Intervention	The intervention consisted of pharmacist applying stickers and criteria for switch to oral antimicrobial therapy to the drug chart. This was compared to the conventional practice of pharmacists reviewing charts and contacting prescribers to discuss an IV-to-PO switch.	Respiratory-relevant antimicrobials/Mixed Infections inclusive of respiratory tract infections	No change in length of stay. Antimicrobial costs reduced by €6.41
Eron, LJ *et al*.^45^	2001	United States of America	Clinical Guidelines and Protocol Implementation	Patients discharged by an infectious disease specialists were compared to patients discharged by an internal medicine physician; antibiotic prescribing patterns were compared.	CAP	Patients cared for by the infectious diseases’ hospitalist had a shorter average length of stay by 1.7 days. Patients treated by the infectious disease hospitalized had a greater rate of IV-to-PO switching.
Omidvari, K *et al*.^46^	1998	United States of America	Clinical Guidelines and Protocol Implementation	Patients were randomized to receive either a conventional course of intravenous antibiotics, or an abbreviated course of 2 days of IV, followed by a switch to PO antibiotics.	CAP	No differences reported in cure rate or survival. Patients who received oral therapy had shorter hospital days by 2 days.
DiDiodato, G *et al*.^47^	2016	Canada	Audit and Feedback Systems/Pharmacist-Led Intervention	A prospective chart audit and physician feedback approach was utilized. An infectious disease-trained pharmacist and physician conducted each intervention	CAP	Length of stay was reduced by 11% Time to antibiotic discontinuation was shortened by 29% There was no change to length of stay.
Jarab, AS *et al*.^48^	2018	Jordan	Audit and Feedback Systems	A retrospective cross-sectional audit was conducted on patients who were prescribed IV antibiotics. Eligibility of IV-to-PO switch was determined based on the Society for Healthcare Epidemiology of America criteria.	CAP/HAP	47.5% were prescribed IV antibiotics despite being eligible for oral conversion.
Walker, RE *et al*.^49^	2023	United States of America	Pharmacist-Led Intervention	An infectious diseases pharmacist provided recommendation for IV-to-PO switches, and descriptive statistics to describe clinician acceptance rates.	CAP	Clinicians were generally receptive to ID pharmacist-led CAP recommendations with an overall acceptance rate of 72%. Prescribers were most receptive to recommendations for IV to PO conversion.
Clark LC *et al*.^50^	2000	United States of America	Clinical Guidelines and Protocol Implementation	Guidelines for the treatment of CAP were introduced, with recommendations including levofloxacin as monotherapy. Length of stay, death rate and cost were monitored.	CAP	Patients on monotherapy levofloxacin had a lower death rate (1.29% versus 7.1%). Total hospital costs were $251 516 less than costs prior to guideline implementation.
Schouten, JA *et al*.^51^	2007	The Netherlands	Clinical Guidelines and Protocol Implementation/Pharmacist-Led Intervention	A clinical pharmacist, microbiologist, pulmonologist and quality improvement officer organized a local organizing committee. Feedback to doctors was presented at these committees in writing. Consensus critical-care pathways were distributed to all doctors as laminated pocket cards. Interventions were then adapted to the needs of doctors perceived barriers.	CAP	The rate of guideline-adherent antibiotic prescribing increased by 14%. IV-to-PO switching improved in the control hospitals (53.3% to 71.9%), than in the intervention hospitals (74% to 83.6%). Timely administration of antibiotics for CAP increased significantly.
Zaal *et al*.^52^	2020	The Netherlands	Pharmacist-Led Intervention	Pharmacists provide recommendations to the prescriber usually over the telephone, with interventions recorded in the patient’s electronic medical record.	Respiratory-relevant antimicrobials/Mixed Infections inclusive of respiratory tract infections	Physicians’ acceptance rates of pharmacist suggestions were reported at 71.2%. Acceptance was significantly associated with the number of prescribed drugs.
Ramirez, JA *et al*.^53^	1999	United States of America	Clinical Guidelines and Protocol Implementation	A preprinted form was used for all hospitalized patients with CAP. Patients were then assessed against the criteria stipulated within the preprinted form by a physician.	CAP	Clinical failure was documented in 1 patient. Early switch and discharge were performed in 44% of patients. At least 95% of patients were satisfied with the care they had received.
Fine, MJ *et al*.^54^	2003	United States of America	Clinical Guidelines and Protocol Implementation	Physician groups were assigned randomly to receive a practice guideline alone or a practice guideline that was implemented using a multifaceted strategy, which included education	CAP	Reduced IV therapy duration (3.0 days versus 4.0 days). No difference in mortality.
Carratela J *et al*.^55^	2012	Spain	Clinical Guidelines and Protocol Implementation	Patients admitted with CAP were randomized to follow a-step critical pathway and use of objective criteria for IV-to-PO antibiotic switching, or usual care.	CAP	Reduction in the length of stay (3.9 days versus 6.0 days). Duration of intravenous antibiotic therapy was lower in the intervention group (2.0 days compared to 4.0 days). Greater incidence of adverse drug reactions in the usual care group (4.5% versus 15.9%).
Feagan BG^56^	2001	Canada	Clinical Guidelines and Protocol Implementation	A pathway was implemented to improve the efficiency of treating CAP.	CAP	Use of a pathway was associated with a 1.7-day reduction in the bed days/patient managed (length of stay)
Yen, YH and Chen, *et al*.^57^	2012	Taiwan	Pharmacist-Led Intervention	A pre- and post-intervention analysis approach was taken. Pharmacists reviewed and intervened on all prescriptions for levofloxacin.	CAP/HAP	The length of hospital stay decreased by 9.1 days. Cost of levofloxacin decreased by $119.9 on average. Total inpatient expenditure also decreased by $2447.
Laing, R.B.S. *et al*.^58^	1998	United Kingdom	Clinical Guidelines and Protocol Implementation	Three different strategies were utilized. One audit was conducted after guideline introduction, the second after guidelines were placed in patient case notes, and the third after guidelines introduced into drug charts.	Respiratory relevant antimicrobials/Mixed infections	Implementing guidelines into drug charts resulted in the shortest duration of IV therapy. There was no difference in the length of stay between patient groups, but patients switched from IV-to-PO spent less time in hospital.
Van Schooneveld, T.C. *et al*.^59^	2020	United States of America	Pharmacist-Led Intervention	Pharmacist-led antibiotic time outs were implemented on 3 medicine teams, whereas the other 3 medicine teams maintained usual care.	Respiratory relevant antimicrobials/Mixed infections	Intravenous antibiotic use decreased following pharmacist-led antibiotic time-out implementation. The ratio of PO-to-IV antibiotics was higher in the usual care group. There were no differences in mortality, length of stay, readmission or antibiotic adverse events.
Ciarkowski Ce *et al*.^60^	2020	United States of America	Clinical Guidelines and Protocol Implementation /Education/Computerized Decision Support System	Phase 1 of the study involved educating clinicians alone. Phase 2 involved educating clinicians and implementing a clinical decision support tool (CDS) with a pathway, coupled with active antimicrobial stewardship.	CAP	Phase 2 was associated with significantly lower length of intravenous and total antibiotic therapy, higher procalcitonin lab utilization, and a 20% cost reduction compared with baseline.
Nguyen, TNT *et al*.^61^	2023	Vietnam	Clinical Guidelines and Protocol Implementation/Pharmacist-Led Intervention	A before-and-after study was conducted amongst inpatients. Guidelines were implemented, and ward pharmacists would give feedback to clinicians based on eligible cases.	Respiratory-relevant antimicrobials/Mixed Infections inclusive of respiratory tract infections	IV-to-PO switches increased by 23.4% during the intervention period. There were no statistically significant differences with regard to the duration of IV therapy, length of hospital stay or treatment outcomes.
Anusha, B *et al*.^62^	2021	India	Pharmacist-Led Intervention	Clinical pharmacists would make recommendations for early conversion of intravenous to oral antibiotics; outcomes were compared to IV-to-PO switches prior to pharmacist involvement. This was done in a retrospective and prospective phase.	Respiratory relevant antimicrobials/Mixed Infections inclusive of respiratory tract infections	The average length of hospital stay was less in the prospective phase (4.4 days) rather than the retrospective phase (6.0 days). The additional number of intravenous therapy days decreased from 112 days retrospectively to 35 days prospectively.
Sze, W.T. *et al*.^63^	2018	Malaysia	Clinical Guidelines and Protocol Implementation	In the pre-intervention phase, pharmacists reviewed medication charts and verbally informed prescribers of eligible IV-to-PO switches. In the post-intervention phase, pharmacists attached a printed checklist which contained IV-to-PO switch criteria to patient’s medical notes, and stickers of IV-to-PO switch were applied to the prescription to serve as reminders.	CAP/HAP	Duration of IV therapy in the post-intervention phase was less than in the pre-intervention phase. Length of hospital stay in the post-intervention phase was shortened by 1.44 days. Antibiotic costs savings increased significantly in the post intervention phase (21.96 versus 13.10).
Tejaswini, YS *et al*.^64^	2018	India	Clinical Guidelines and Protocol Implementation	IV-to-PO switching was observed in practice according to pre-defined criteria for clinical stability.	Respiratory-relevant antimicrobials/Mixed Infections inclusive of respiratory tract infections	IV-to-PO switching was associated with a lower length of stay (6.84 days compared to 8.71 days).
Engel MF *et al*.^65^	2014	The Netherlands	Clinical Guidelines and Protocol Implementation/Education	Interactive educational sessions were conducted with medical specialists, residents and nurses. A switch protocol was presented with nurses asking doctors to switch. Laminated pocket cards were put in the personal mailboxes of all participants, to remind clinicians of the need to switch.	CAP	Reduced IV therapy duration by 4 days.
Fésüs A *et al*.^66^	2024	Hungary	Clinical Guidelines and Protocol Implementation	Written and published guidelines were made available to clinicians, as well as offering continuous supervision and counselling services on antibiotic therapy.	CAP	Antimicrobial Stewardship programs were associated with a reduction in length of stay (7.09 days compared to 8.85 days).
Kuti, JL *et al*.^67^	2002	United States of America	Pharmacist-Led Intervention	Pharmacists used predetermined clinical criteria to convert levofloxacin therapy from IV to PO antibiotics without physician approval.	CAP	Clinical success rate was rated at 100%. Reduced length of stay (6 versus 9.5 days). Reduction in institutional healthcare costs ($13 931 versus $17 198).
Fischer, MA *et al*.^68^	2003	United States of America	Computerized Decision Support System	Computerized order entry systems were used to prompt physicians to convert appropriate intravenous medications to the oral route	Mixed Infections inclusive of respiratory tract infections/Respiratory relevant antimicrobials	The average intravenous defined daily dose declined by 11.1%. Average oral defined daily dose increased by 3.7%. Total monthly use of intravenous preparations of all targeted medications declined in the 4 months after the intervention began.
Golali, E. *et al*.^69^	2019	Iran	Pharmacist-Led Intervention	Medical files of all patients receiving intravenous antibiotics were reviewed by a clinical pharmacy specialist.	CAP/HAP	Acceptance rates of clinical pharmacy recommendations by physicians were 80.2%. Hospital length of stay reduced from 16.1 days to 11.6 days pre- and post-intervention. There was no difference in mortality rate.
Halley HJ^70^	2000	United States of America	Pharmacist-Led Intervention	A pathway was developed by a multidisciplinary team and approved by pharmacy. This was then implemented; patients receiving antimicrobial therapy for CAP were targeted for inclusion in the clinical pathway and compared with non-pathway patients.	CAP	Pathway patients had a 1.2-day shorter length of stay than non-pathway patients. Projected drug cost savings were deemed to be more than $22 000 annually.
Davis SL *et al*.^71^	2005	United States of America	Pharmacist-Led Intervention	Patients with CAP were subject to different pharmacy interventions (PI): Standard of care, an IV-to-PO switch advised by a pharmacist, and an IV-to-PO switch initiated by a pharmacist automatically.	CAP	Duration of IV antibiotics decreased by 1.18 days. f Antibiotic costs were reduced by $110 per patient. Length of stay was similar for all patients.
Sadeq, A.A. *et al*.^72^	2021	United Arab Emirates	Pharmacist-Led Intervention/Multidisciplinary Team Approaches	Clinical pharmacists reviewed patient cases and made recommendations. Complex cases were escalated to the infectious diseases clinical pharmacist and physicians when required.	Respiratory-relevant antimicrobials/Mixed Infections inclusive of respiratory tract infections	In the intervention group, a significant reduction in length of hospital stay, and rate of readmission was reported. IV antibiotic usage decreased by a ratio of 0.7 in the intervention period.
Peyrani P.^73^	2013	Spain	Clinical Guidelines and Protocol Implementation	Patients were randomly assigned to a three-step pathway or received usual care.	CAP	Median length of stay was 2.1 days less in the pathway arm than the usual care arm. Duration of intravenous antibiotic therapy was 2 days less in the pathway arm.
Paediatric Populations						
In-iw, S *et al*.^74^	2015	Thailand	Clinical Guidelines and Protocol Implementation	Randomized patients to either receive an IV-to-PO switch of antibiotics or receive the standard regimen of intravenous antibiotic therapy	CAP	Significant reduction in Length of Stay in the switch therapy group (3.81 days versus 4.77 days). No increase in morbidity and/or mortality.
Al-Eidan, F.A. *et al*.^75^	1999	Ireland	Clinical Guidelines and Protocol Implementation	A guideline was created to stipulate criteria under which paediatric patients would be switched from IV to PO.	Respiratory relevant antimicrobials/Mixed Infections inclusive of respiratory tract infections	Those switched from IV-to-PO had a shorter Length of Stay (4.0 days versus 8.3 days). Reduction in total IV administration (1.7 days versus 5.6 days). Reduction of healthcare costs by 52%.
Kawamura, M *et al*.^76^	2017	Japan	Clinical Guidelines and Protocol Implementation	A paediatric infectious disease specialist directed clinical interventions to make IV-to-PO switches against standardized guidelines.	CAP	Length of hospital stay in those patients who were switched from IV-to-PO was shorter (6 days) than those who were not switched (10 days). There were no patients requiring rehospitalization.
Adult and Paediatric Mixed Populations						
Shindo Y *et al*.^77^	2008	Japan	Clinical Guidelines and Protocol Implementation	A clinical pathway (CP) was implemented with standard guidance as to when patients should be switched from an IV beta-lactam to an oral fluoroquinolone. Patients were divided into two groups: 1. Patients with a CP 2. Patients who were non-CP.	CAP	The CP group was associated with significant reductions or a decrease in LOS (10.38 days to 6.65 days), hospital charges (¥302 255 to ¥214 170), time taken to attain clinical stability (8.45 days to 5.50 days), and duration of intravenous antibiotic treatment in the mild and moderate classes (7.76 days to 4.04 days).
Melo Rodrigues, *et al*.^78^	2013	Brazil	Clinical Guidelines and Protocol Implementation	Guidelines were written by two infectious disease physicians and one pharmacist and then implemented.	Respiratory relevant antimicrobials/Mixed Infections inclusive of respiratory tract infections	Sequential antibiotic therapy only occurred in 4 and 5 courses of treatment. There was no difference in IV antibiotic usage in the PIP and IP periods.
Maripuu, H *et al*.^79^	2014	United Kingdom	Audit and Feedback Systems	The audit involved collecting data on admitted patients diagnosed with either lower respiratory tract infections or urinary tract infections who subsequently received antibiotic treatment.	CAP/HAP/Mixed Infections (UTIs)	The percentage adherence rate for hospital antibiotic policy was 68%. Documentation of CURB-65 score was found in 80% of the patients’ notes. 58.5% of patients were switched from IV-to-PO in accordance with the policy.
Di Giammarino, L. *et al*.^80^	2005	Switzerland	Clinical Guidelines and Protocol Implementation/Audit and Feedback Systems	An observational approach was taken to check adherence to guidelines.	CAP	47% of patients had a delayed switch from IV to PO, with 240 days of cumulative delay.

### Clinical guidelines and protocol implementation

Most studies evaluated guideline or protocol implementation, with effectiveness varying due to implementation strategy. Several randomized controlled trials demonstrated the efficacy of protocol-driven IV-to-PO switching. Clinical Guideline and Protocol Implementation were the only type of intervention in which paediatric populations were examined.

#### Studies with adult populations

Oosterheert *et al*. conducted a computer-randomized trial in the Netherlands where patients were assigned to receive either protocol-guided IV-to-PO switching or standard IV therapy for community-acquired pneumonia (CAP).^27^ The intervention reduced IV antibiotic duration by 3.4 days and shortened hospital length of stay (LOS) by 1.9 days, whilst maintaining comparable cure rates (83% versus 85%). Castro-Guardiola *et al*. implemented similar criteria-based switching in Spain, randomizing patients to early-switch (once afebrile) versus standard oral therapy.^32^ The early-switch group experienced fewer adverse events (16% versus 34%) and shorter LOS (6 days versus 11 days), generating cost savings primarily attributed to reduced hospitalization. Similarly, Carratala *et al*. implemented a step-down critical pathway in Spain using objective criteria for switching, which reduced LOS from 6.0 to 3.9 days and IV therapy duration from 4.0 to 2.0 days, whilst simultaneously reducing adverse drug reactions (15.9% to 4.5%).^55^

Studies that examined compliance with guidelines and subsequent guideline implementation revealed variation in adherence and associated clinical outcomes. Frei *et al*. stratified patients based on American Thoracic Society (ATS) guideline concordance, finding that guideline-concordant care was associated with quicker IV-to-PO switching (4.5 versus 5.9 days), shorter LOS (5.0 versus 6.2 days), and lower in-hospital mortality (3% versus 7%).^36^ The study also demonstrated quicker time to clinical stability in concordant cases (4% versus 1%). Mazzola *et al*. found that only 66% of patients received guideline-recommended initial antibiotics, route, and dose.^37^

#### Studies with adult populations that included mixed infection types

Some studies examined respiratory-relevant antimicrobials across mixed infection types. Shrayteh *et al*. documented that among 452 IV antibiotic courses where patients were eligible for conversion, only one-third were switched, with the remainder continuing IV therapy beyond day 3 despite meeting criteria.^17^

Buyle *et al*. conducted a three-phase intervention study in Belgium comparing progressively intensive implementation methods.^40^ Phase 1 involved guideline dissemination to all prescribers, resulting in patients receiving IV antibiotics 4.1 days longer than necessary, with mean additional costs of €188. Phase 2 added interactive educational sessions delivered by infectious disease specialists, reducing unnecessary IV days to 3.5 and costs to €103. Phase 3 incorporated pharmacist-led interventions with pre-printed IV-to-PO switch reminders attached to patient charts, further reducing unnecessary IV days to 1.0 and costs to €44. This progressive improvement demonstrated that workflow integration can transform guideline effectiveness by embedding recommendations at critical decision points. Laing *et al*. demonstrated this through progressive implementation phases^58^: guidelines placed passively in case notes achieved no IV duration reduction versus baseline, as clinicians needed to remember guidelines existed and actively seek them during prescribing decisions. However, integration into medication administration charts, which were reviewed mandatorily during daily prescribing rounds, reduced duration to 3.7 days (*P* < 0.05), a 16% improvement. Fine *et al*. (2003) compared guideline provision versus guideline implementation using a multifaceted strategy including education. The multifaceted approach achieved reduced IV therapy duration (3.0 days versus 4.0 days) with no difference in mortality, whilst guideline provision showed minimal effect. Similarly, Hagaman *et al*. documented that physician awareness and guideline usage increased from 5% to 40% following active implementation efforts, with corresponding increases in switch therapy rates (60% to 86%) and LOS reductions of 1.2 days.^41^ This contrast illustrated that guideline effectiveness depends less on recommendation quality and more on delivery mechanism: passive guidelines function as weak interventions relying on individual memory, whilst workflow-integrated guidelines approximate administrative or engineering controls by making switching criteria visible and actionable at the precise moment decisions occur.

Economic analyses consistently demonstrated cost savings associated with guideline implementation. Emerson *et al*. estimated annual cost savings of nearly $300 000 following CAP guideline implementation in the United States, coupled with 1-day LOS reductions.^30^ Clark *et al*. reported total hospital cost reductions of $251 516 following guideline introduction recommending levofloxacin monotherapy, which was also associated with lower mortality rates (1.29% versus 7.1%).^50^ Feagan documented that pathway use in Canada was associated with a 1.7-day reduction in bed days per patient managed.^56^

#### Studies with paediatric populations

In-Iw *et al*. randomized paediatric CAP patients in Thailand to receive either IV-to-PO switching or standard IV therapy.^74^ The intervention achieved significant LOS reduction (3.81 versus 4.77 days) with no increase in morbidity or mortality. Similar outcomes were reported by Al-Eidan and colleagues, who created guidelines that stipulated criteria for switching paediatric patients from IV to PO in Ireland.^75^ Patients who had been switched from IV-to-PO antibiotics had shorter LOS (4.0 versus 8.3 days), reduced total IV administration (1.7 versus 5.6 days), and 52% reduction in healthcare costs. Kawamura *et al*. evaluated early IV-to-PO antibiotic switching for lower respiratory tract infections in paediatric patients with severe motor and intellectual disabilities in Japan.^76^ A paediatric infectious disease specialist directed clinical interventions using standardized switching guidelines. Patients switched from IV-to-PO had significantly shorter hospital stays (6 days) compared to those maintained on IV therapy (10 days), with no patients requiring rehospitalization.

#### Studies with mixed populations

Shindo *et al*., implemented a clinical pathway in Japan specifying standardized guidance for IV-to-PO switching from beta-lactams to fluoroquinolones.^77^ The pathway group achieved significant reductions in LOS (10.38 days to 6.65 days), hospital charges (¥302 255 to ¥214 170), time to clinical stability (8.45 to 5.50 days), and IV antibiotic duration in mild-moderate cases (7.76 days to 4.04 days). Similarly, Melo Rodrigues *et al*. implemented guidelines for sequential antibiotic therapy in a Brazilian university hospital.^78^ Guidelines were developed by two infectious disease physicians and one pharmacist and subsequently implemented. Despite guideline introduction, sequential antibiotic therapy occurred in only four treatment courses pre-implementation and five courses post-implementation. No significant difference in IV antibiotic usage was observed between periods. Di Giammarino *et al*. conducted an observational evaluation of antibiotic prescription practices in a Swiss hospital.^80^ An observational approach was taken to check adherence to guidelines for antibiotic use and IV-to-PO switching. The audit revealed that 47% of patients had a delayed switch from IV to PO antibiotics, with 240 days of cumulative delay across all patients.

### Pharmacist-led interventions

Twenty-one studies evaluated ‘pharmacist-led interventions,’ which varied considerably in design, and the extent to which pharmacists were involved. Studies that examined pharmacist-led interventions included adult populations. No paediatric-specific pharmacist-led interventions were identified.

#### Retrospective chart review

Some studies employed retrospective chart review; in these studies, pharmacists identified patients meeting switching criteria through daily review of medication administration records and microbiology results, then communicated recommendations to prescribing physicians for final approval. This approach was used by Xu and colleagues, with clinical pharmacists reviewing all patients receiving IV antibiotics and generating written recommendations placed in patient charts.^35^ The intervention was combined with computerized reminders sent to physician smartphones when patients met switching criteria. Mean IV therapy duration reduced from 6.75 days to 5.52 days. Physician acceptance of pharmacist recommendations was 78%, with main reasons for rejection being physician preference for longer IV courses (62% of rejections) and concerns about gastrointestinal tolerance (38% of rejections). Zaal *et al*. employed a similar model in the Netherlands, where pharmacists provided recommendations to prescribers, usually over the telephone, with interventions recorded in the patient's electronic medical record.^52^ Physicians’ acceptance rates of pharmacist suggestions were reported at 71.2%, with acceptance significantly associated with the number of prescribed drugs. Shrayteh *et al*. (2014) employed clinical pharmacists to conduct prospective daily audit of all patients receiving IV antibiotics in general medical wards who were eligible for IV-to-PO switches.^17^ Pharmacists would then provide verbal feedback to prescribing physicians, using standardized switching criteria. This intervention increased switching rates from 34% to 58% and reduced mean IV duration from 5.8 to 3.2 days (*P* < 0.0001), though mean length of stay (LOS) remained unchanged.

#### Visual reminders and stickers

Several studies incorporated visual prompts and stickers applied directly to patient charts or prescription records by pharmacists to enhance visibility of switching recommendations. Dunn *et al*. compared two approaches in Ireland: conventional pharmacist chart review with verbal communication to prescribers versus pharmacists applying stickers with IV-to-PO switch criteria directly onto drug charts.^4^ The sticker intervention demonstrated no change in LOS but achieved antimicrobial cost reductions of €6.41 per patient. Sze *et al*. implemented a similar strategy in Malaysia where, in the pre-intervention phase, pharmacists reviewed medication charts and verbally informed prescribers of eligible switches.^63^ In the post-intervention phase, pharmacists attached printed checklists containing IV-to-PO switch criteria to patients’ medical notes and applied stickers to prescriptions as reminders. The post-intervention approach achieved shorter IV therapy duration, reduced LOS by 1.44 days, and significantly increased antibiotic cost savings (21.96 versus 13.10).

#### Prospective interventions with ward round participation

Other studies involved pharmacists participating in ward rounds or conducting real-time review during active decision-making periods. Yen *et al*. implemented prospective pharmacist involvement in a Taiwanese teaching hospital where clinical pharmacists joined daily multidisciplinary rounds, reviewed patients receiving IV antibiotics, and made verbal recommendations directly to attending physicians with immediate discussion of concerns.^57^ This study reported reductions in mean hospital stay from 27.2 days to 16.1 days and reduced the length of IV antibiotic treatment days from 8.3 days to 6.6 days. Physician acceptance rates of pharmacist recommendations reached 89% with the face-to-face communication model, substantially higher than the 65% acceptance previously achieved with written recommendations in the same institution. Grill *et al*. implemented a model in Germany where ward pharmacists judged appropriateness of antimicrobial utilization within 24 hours of therapy initiation.^26^ This intervention achieved a reduction in overall antimicrobial course length (10 days versus 11 days), reduced IV antibiotic duration (8 days versus 10 days), and decreased organizational antimicrobial costs by 16%. Van Niekerk *et al*. integrated pharmacists into ward rounds in South Africa over a 7-week period using developed guidelines.^29^ The intervention reduced IV therapy duration from 7.2 days to 5.2 days, shortened LOS (11.5 days versus 10.7 days), and demonstrated cost savings of £1414.41.

#### Autonomous pharmacist-initiated switching protocols

Several studies granted pharmacists autonomous authority to initiate IV-to-PO switches without requiring prior physician approval. Kuti *et al*. employed this approach in a US academic medical centre where infectious diseases-trained clinical pharmacists automatically converted intravenous fluoroquinolones to oral formulations when patients met specific criteria.^67^ This protocol reduced average conversion treatment time by 4 days whilst maintaining 100% cure rates for patients. Only 2.4% of patients required return to IV therapy, all for non-infectious complications such as nausea precluding oral intake or development of ileus. Physician override of automatic conversions occurred in 8% of cases, primarily for patients with complicated infections or concerns about absorption. Davis *et al*. compared three different pharmacy intervention models in the United States: standard care, pharmacist-advised IV-to-PO switches requiring physician approval, and pharmacist-initiated IV-to-PO switches without prior approval.^71^ The autonomous pharmacist-initiated protocol reduced IV antibiotic duration by 1.18 days and antibiotic costs by $110 per patient, with similar LOS across all groups. Hendrickson *et al*. implemented a protocol where clinical pharmacists reviewed patients for step-down therapy eligibility using specific criteria, then initiated switches autonomously.^34^ This approach reduced total antimicrobial therapy duration (9.1 days versus 11.9 days) and achieved cost savings of $46.05 per patient.

Reported limitations of pharmacist-led interventions involved how long the effect of the intervention lasted after withdrawal of active implementation. Van Niekerk *et al*. documented that during the active intervention period with daily pharmacist review and recommendations, switching rates increased from 16% at baseline to 43.9% (*P* < 0.0005). However, at 3 months following withdrawal of dedicated pharmacist time when pharmacists returned to other duties, switching rates declined to 20.8%, approaching baseline levels.^29^ Another limitation was a lack of consistency throughout the week. Analysis of switching timing revealed that patients admitted Thursday-Saturday experienced delays in switching compared to Monday-Wednesday admissions (mean delay 1.8 days, *P* = 0.03), attributed to lack of weekend pharmacist coverage. The study calculated that extending pharmacist coverage to 7 days per week would require 40% additional pharmacist full-time equivalents, raising questions about cost-effectiveness of continuous coverage versus accepting modest delays for weekend admissions.

### Education-based interventions

Seven studies incorporated educational components as part of their interventions to promote IV-to-PO antibiotic switching. Educational interventions varied substantially in delivery method, target audience, content focus, and degree of tailoring to identified barriers. Studies that evaluated education-based interventions only included adult populations.

#### Interactive educational sessions led by specialists

Buyle and colleagues conducted interactive educational sessions that were led by infectious disease (ID) specialists.^40^ In these sessions, ID specialists taught medical staff, predominantly resident doctors, about the similarities in bioavailability between intravenous and oral preparations of antimicrobials, as well as differences in cost between the preparation. These educational sessions reduced the duration of unnecessary IV antibiotic therapy by 3.5 days and decreased mean additional costs associated with IV antibiotic therapy from €188.0 to €103.0. However, adding pharmacist-led interventions with visual reminders in phase 3 further improved outcomes, reducing unnecessary IV days to 1.0 and costs to €44.0, suggesting that education alone achieved intermediate rather than optimal results. A similar approach was taken by Engel and colleagues, who conducted interactive educational sessions with medical specialists, residents, and nurses in the Netherlands.^82^ The educational content presented a switch protocol, with nurses designated to prompt doctors to switch. Laminated pocket cards, which detailed pathways and IV-to-PO antibiotic switch criteria, were also placed in personal mailboxes of all participants as reminders. IV therapy duration decreased by 4 days following the intervention.

Ciarkowski and colleagues implemented a two-phase intervention in the United States of America. Phase 1 involved educating clinicians alone. Phase 2 combined clinician education with implementation of a clinical decision support tool containing a care pathway, coupled with active AMS team involvement. Phase 2 was associated with significantly lower length of intravenous and total antibiotic therapy, higher procalcitonin utilization, and a 20% cost reduction compared with baseline.

#### Barrier-targeted educational interventions

Other education-based interventions targeted specific barriers known to educators that would prevent IV-to-PO antibiotic switches. Schouten *et al*. employed a needs assessment approach in the Netherlands to identify specific physician concerns impeding IV-to-PO switching prior to designing educational content.^51^ The assessment revealed four primary barriers: concerns about oral bioavailability in severely ill patients (72% of physicians), uncertainty about oral dosing equivalents (58%), medicolegal risk perceptions (41%), and clinical inertia (38%). Educational sessions led by infectious disease (ID) specialists were subsequently tailored to address these specific barriers through evidence-based discussion of pharmacokinetic data, legal precedents, and institutional protocols. This targeted approach, combined with feedback mechanisms increased guideline-adherent antibiotic prescribing by 14%. IV-to-PO switching rates improved (74% to 83.6%) and timely administration of antibiotics for CAP also increased significantly. These findings suggested that educational interventions achieved greater effectiveness when tailored to audience-specific concerns and integrated with ongoing feedback mechanisms, rather than delivering standardized content uniformly across all clinician groups. In the case of the narrative that some physicians have doubts regarding the efficacy of oral antimicrobials in treating pleural infections, tailoring interventions to incorporate respiratory elements may improve outcomes.

Ahmed *et al*. implemented education coupled with guideline introduction and antibiogram development in India for ventilator-associated pneumonia (VAP) management.^44^ The educational intervention improved multiple antimicrobial stewardship metrics: the proportion of patients undergoing antibiotic de-escalation increased from 21% to 36%, appropriate antibiotic use improved from 25% to 46%, and IV-to-PO switching rates increased. These improvements translated to reduced expenditure (₹24 705.5 pre-intervention versus ₹16 517.5 post-intervention), demonstrating that education targeted at specific infection syndromes with supporting tools could achieve substantial clinical and economic benefits.

#### Educational interventions with computer decision support systems

Mouwen *et al*. implemented a multimodal intervention in the Netherlands combining physician education with multiple reinforcement mechanisms.^16^ Physicians received educational sessions on guideline-based IV-to-PO switching criteria. Additionally, the intervention incorporated electronic notifications through the patient management system that alerted physicians when individual patients met switching criteria based on documented clinical parameters. This technologically augmented educational approach reduced IV antibiotic duration by 2 days and decreased hospital LOS by 1 day. The combination of initial education and automated patient-specific electronic prompts appeared to address both knowledge gaps and workflow integration challenges simultaneously. Lesprit *et al*. implemented a similar approach in France using computer-generated listings that prompted physicians to review antibiotic prescriptions.^43^ Two strategies were compared: electronic prompts alone versus electronic prompts coupled with infectious disease physician consultation and educational feedback. Significantly more prescriptions were modified when infectious disease physician advice and education followed electronic prompts compared to prompts issued independently. This finding underscored that technology-delivered reminders achieved greater impact when combined with expert consultation and just-in-time education rather than serving as standalone interventions.

### Computerized decision support systems

Five studies evaluated computerized decision support systems (CDSS) as part of interventions to promote IV-to-PO antibiotic switching. All CDSS interventions incorporated electronic alerts, reminders, or prompts integrated into existing clinical workflows, though implementation approaches and integration depth varied substantially.

Fischer *et al*. embedded prompts directly into prescribing systems in the United States.^68^ The prompts appeared automatically when clinicians prescribed specific IV medications that had oral alternatives available. This intervention achieved an 11.1% reduction in the average IV defined daily doses (*P* = 0.002) and a 3.7% increase in oral doses (*P* = 0.002).^68^ Total monthly use of IV preparations for all targeted medications declined in the 4 months following intervention implementation. Lesprit *et al*. also implemented computer-generated prescription screening in France that prompted physicians to review antibiotic prescriptions. Ciarkowski *et al*. evaluated clinical decision support linked with pathways, achieving significantly lower IV and total antibiotic durations, higher procalcitonin utilization, and 20% cost reductions. Integration with pathway protocols and education appeared essential to effectiveness.^60^

Studies reporting physician acceptance rates or alert response rates demonstrated substantial variation. Xu *et al*. reported 78% acceptance of pharmacist recommendations delivered via the CDSS-augmented system. Lesprit *et al*. found that physicians frequently continued IV therapy despite CDSS prompts, though modification rates improved when ID consultation accompanied electronic alerts. Fischer *et al*. demonstrated measurable prescribing behaviour change through aggregate data on IV versus oral prescribing patterns but did not report individual alert acceptance rates. No studies explicitly measured alert fatigue, override rates stratified by clinical appropriateness, or long-term sustainability of CDSS effectiveness beyond immediate post-implementation periods but could be used to explain the lack of responsiveness to CDSS warnings offered to end-users.

### Audit and feedback systems

Audit and feedback address a key mechanism of educational intervention decay identified in aforementioned studies^83^; without performance data, clinicians lack information about whether they maintain improved practices or have regressed. By making prescribing patterns visible and accountable, feedback mechanisms can be used towards administrative controls that create organizational pressure for compliance. The effectiveness of audit and feedback varied substantially based on implementation characteristics, particularly whether feedback was individualized to prescribers versus presented as aggregate institutional data. Studies that examined audit and feedback systems included both adult populations and mixed populations (adult and paediatric).

#### Studies with adult populations

Golali and colleagues documented pharmacist-led audit processes in Iran, identifying most common recommendations as discontinuing antibiotics (35%) and IV-to-oral switching (22%), with 80.2% physician acceptance rates.^69^ Crucially, guideline discrepancies significantly reduced between pre-intervention and late intervention periods, suggesting audit created accountability mechanisms sustaining improvement. Fok and colleagues revealed baseline performance gaps through a comprehensive audit, providing data to justify targeted interventions and establish metrics for subsequent comparison.^39^ Buyle and colleagues demonstrated that ongoing measurement and feedback achieved 3.3% reduction in IV fluoroquinolone consumption, with sustained monitoring maintaining accountability over time.^40^ Studies consistently found individualized feedback to prescribers achieved greater engagement than aggregate institutional data alone, as personalized data created direct accountability and enabled self-assessment against peers, whilst institutional averages allowed individual clinicians to assume problems lay elsewhere. This supported the principle that effective behaviour change interventions must provide timely, specific, actionable feedback directly relevant to the individual decision-maker.

#### Studies with mixed populations

Maripuu and colleagues conducted an audit in a United Kingdom based hospital examining antibiotic use among patients admitted with lower respiratory tract infections or urinary tract infections.^79^ The review assessed adherence to local antibiotic guidelines, documentation of illness severity, and IV-to-oral switch practices. The audit found that 68% of prescriptions followed hospital policy, CURB-65 scores were recorded in 80% of cases, and 58.5% of eligible patients were appropriately switched from IV to oral therapy. These results highlighted room for improvement, adherence to antibiotic IV-to-PO switch policy and better documentation of the CURB-65 score in patients’ notes.

### Multidisciplinary team approaches

Studies that focused on employing multidisciplinary team approaches were conducted with adult populations. One study was explicitly categorized as employing a multidisciplinary team approach, though several other interventions incorporated collaborative elements involving multiple healthcare professional groups working in coordinated fashion. Multidisciplinary models can address multiple mechanisms of behaviour change simultaneously, by distributing cognitive workload across team members with complementary expertise. In this respect, they have the capacity to encourage interprofessional workflow rather than relying on individual physician initiative and create peer accountability through collaborative review processes. Sadeq *et al*. implemented a tiered multidisciplinary approach in the United Arab Emirates, where clinical pharmacists conducted initial patient case reviews and made recommendations for IV-to-PO switching. Complex cases that exceeded pharmacist scope or required specialist expertise were escalated to infectious disease-trained clinical pharmacists and infectious disease physicians for collaborative decision making. This structured escalation model demonstrated significant reductions in LOS (*P* < 0.01), readmissions (*P* < 0.01), and mortality (*P* < 0.01).^72^ Halley *et al*. developed a clinical pathway in the United States through formal multidisciplinary team collaboration involving physicians, pharmacists and other healthcare professionals. Pathway patients experienced 1.2-day shorter LOS compared to non-pathway patients. Projected annual drug cost savings exceeded $22 000. The multidisciplinary development process appeared to enhance pathway acceptability and implementation fidelity, though the study did not explicitly measure team functioning or professional group-specific contributions to outcomes. Eron and colleagues compared CAP management outcomes between infectious disease specialists and internal medicine physicians in the United States, examining whether specialist expertise influenced IV-to-PO switching practices and associated clinical outcomes. Eron and colleagues showed infectious disease specialists achieved shorter LOS (mean difference 1.7 days), no readmissions, and higher satisfaction versus internal medicine hospitalists, with earlier IV to oral switching for CAP.^45^ This may suggest that specialized training in antimicrobial management influenced prescribing behaviour. This specialist-versus-generalist comparison highlighted potential benefits of infectious disease expertise but simultaneously revealed practical limitations: infectious disease specialist availability remains constrained in many healthcare settings, particularly smaller hospitals and rural facilities, necessitating alternative models that distribute antimicrobial expertise through interprofessional collaboration rather than specialist-dependent pathways.

Van Schooneveld *et al*. evaluated pharmacist-led antibiotic time-outs in 1086 patients. Although categorized as a ‘pharmacist-led intervention,’ the time-out process involved structured interprofessional communication where pharmacists reviewed antibiotic appropriateness and engaged physicians in collaborative decision-making. This approach achieved 73.2% early compliance with switching.^59^ However, limited infectious disease specialist availability necessitated alternative models leveraging generalist physicians with pharmacist support. This pragmatic approach recognized that whilst specialist involvement optimized outcomes, structural constraints required scalable models distributing expertise through interprofessional collaboration rather than specialist dependent.

### Multiple intervention combinations

Sixteen studies implemented multiple intervention types simultaneously rather than single-component approaches. These combination interventions demonstrated generally superior outcomes compared to single-component strategies, though attributing effectiveness to specific intervention elements remains challenging.

#### Most common combination: pharmacist-led interventions with clinical guidelines

Six studies combined pharmacist-led interventions with clinical guideline or protocol implementation, representing the most frequently employed multi-component strategy.

Buyle *et al*. implemented a multi-country intervention across Germany, Austria, and Belgium where appropriateness of IV antibiotic administration was reviewed by physicians or pharmacists trained in antimicrobial stewardship, applying pre-defined switching criteria from established guidelines.^28^ The combined approach identified that 37% of IV antibiotic prescribing cases were deemed inappropriate on average, suggesting substantial potential for IV prescribing reduction. Van Niekerk *et al*. developed switching guidelines in South Africa that were subsequently integrated into ward rounds through active pharmacist participation over a 7-week intervention period.^29^ The combined guideline-plus-pharmacist model reduced IV therapy duration from 7.2 days to 5.2 days, shortened LOS from 11.5 days to 10.7 days, and generated cost savings of £1414.41. However, the study also revealed that switching rates declined substantially after withdrawal of dedicated pharmacist resources, from 43.9% during active intervention to 20.8% at 3-month follow-up. Hendrickson *et al*. implemented a protocol in the United States where clinical pharmacists reviewed patients against specific step-down eligibility criteria, then made recommendations to prescribing physicians.^34^ This combined protocol-plus-pharmacist approach reduced total antimicrobial therapy duration from 11.9 days to 9.1 days and achieved cost savings of $46.05 per patient.

#### Triple-component interventions: guidelines, education, and technology

Three studies implemented guidelines, education, and either technology or enhanced pharmacist involvement simultaneously.

Buyle *et al*. demonstrated clear dose–response effects through progressive three-phase implementation in Belgium.^40^ Phase 1 (guideline dissemination alone) resulted in patients receiving IV antibiotics 4.1 days longer than necessary with healthcare associated costs of €188.0 on average. Phase 2 (adding education) reduced unnecessary IV days to 3.5 and reduced healthcare associated costs to €103.0. Phase 3 (adding pharmacist reminders) further reduced unnecessary IV days to 1.0 and reduced costs to €44.0, demonstrating stepwise improvement with each component addition. Ciarkowski *et al*. similarly showed that education alone (Phase 1) achieved minimal impact, whilst combining education with clinical decision support tools, care pathways, and active AMS team involvement in Phase 2 achieved significantly lower IV and total antibiotic duration.^60^ Mouwen *et al*. combined physician education and electronic notifications in the Netherlands, reducing IV duration by 2 days and LOS by 1 day.^16^

#### Comparative effectiveness of combination interventions

Studies that progressively added additional elements to the interventions studied performed better than interventions with singular elements. Buyle *et al*. demonstrated stepwise improvement as components were added: guideline dissemination alone achieved minimal impact, adding education improved outcomes moderately, and incorporating pharmacist involvement with visual reminders achieved optimal results.^40^ Similarly, Ciarkowski *et al*. showed that education alone produced less benefit whilst adding decision support technology and active stewardship dramatically improved outcomes.^60^ Interventions that reported positive clinical outcomes for patients and healthcare associated cost savings appeared to address multiple barriers simultaneously: knowledge gaps through education, decision-making support through guidelines or technology, visibility and accountability through pharmacist involvement, and workflow integration through structured processes. However, no studies explicitly measured the relative contribution of individual components within multi-component interventions. The use of more than one intervention appears to have better outcomes but makes it more challenging to determine whether some interventions are ‘better’ than others.

## Discussion

This systematic review identified 58 studies that evaluated interventions to promote IV-to-PO antibiotic switching. Clinical guideline and protocol implementation was the most frequently employed strategy. Multiple intervention types were used simultaneously in 16 studies. Most interventions demonstrated reductions in IV antibiotic duration of 1–4 days. Hospital length of stay decreased by 1–2 days in most studies reporting this outcome, with larger reductions in some studies. Cost savings were consistently reported, though the magnitude varied substantially by intervention type and healthcare setting. Clinical safety outcomes, including mortality rates and clinical cure rates, remained comparable between intervention and control groups across most studies. Whilst multiple studies reported have shown that early IV-to-PO switching as an antimicrobial stewardship intervention can be used to manage patients with pneumonia, our review has demonstrated that current interventions are not equal in achieving the same clinical and economic outcomes.

### Temporal applicability and the evolution of antimicrobial stewardship practices, and implications for contemporary practice

The studies included in this review were reported from the years 1995 to 2024, during which the clinical, organizational, and pharmacological context for pneumonia management and antimicrobial stewardship have changed. Specific changes over this period include the introduction and establishment of AMS programmes within hospitals,^2,84^ the embedding of pharmacist roles within institutional AMS structures,^85,86^ introduction of infection management guidelines within organizations, the promotion of IV-to-PO switches through national programmes,^85,87,88^ and the progressive de-escalation of fluoroquinolone prescribing.^89,90^ Another notable change includes an increased focus on the identification and management of sepsis, which is of particular relevance given the frequency of sepsis secondary to community-acquired pneumonia.^91–93^ This period saw a substantial shift from paper-based to electronic prescribing systems, which reflects studies using paper-based and electronic systems included this study. Therefore, it is important to consider these changes when applying them to contemporary practice.

Earlier studies in this review predate the formalization of AMS, one key element of which is the development of clinical guidelines to standardize and drive antimicrobial prescribing decisions. The Infectious Diseases Society of America and Society for Healthcare Epidemiology of America (IDSA/SHEA) did not publish comprehensive AMS guidelines until 2007,^94^ with subsequent updates in 2016^95^ and 2024.^96^ These guidelines are relevant beyond a North American context, as IDSA/SHEA recommendations have been widely adopted internationally and have informed AMS frameworks in many of the countries represented in this review. Similarly in the United Kingdom, the consensus for IV-to-PO switching was first published in the United Kingdom in 2023.^7^ Studies conducted before the 2007 IDSA/SHEA guidelines,^87,97,98^ such as those by Hendrickson *et al*. and Laing *et al*., were therefore undertaken in the absence of formalized AMS programme standards; and whether later studies operated within dedicated AMS structures is not consistently reported across the included papers. This represents a contextual limitation of the review, and findings from earlier studies should be interpreted with this in mind. Nonetheless, the 2016 guidelines published by the IDSA/SHEA explicitly consolidated the role of pharmacists in antimicrobial optimization.^95^

Antimicrobial agents that are recommended for pneumonia management have changed over the review period, driven by emerging resistance patterns, new evidence on comparative effectiveness, and changing safety profiles. Several earlier studies evaluated fluoroquinolone-based regimens, such as Clark *et al*. who examined levofloxacin monotherapy, and Kuti *et al*. who evaluated pharmacist-initiated fluoroquinolone switching. Contemporary guidelines now recommend more restrictive fluoroquinolone use due to safety concerns, such as cardiovascular toxicity that can be driven by QT prolongation and aortic aneurysm,^99^ tendinopathy and neuropsychiatric events^100–102^; other concerns include Clostridioides difficile infection risk,^103–105^ and increasing resistance rates.^105^ These safety concerns are reflected in multiple MHRA Drug Safety Updates issued between 2012 and 2019.^106,107^ Despite these changes in specific agents, the fundamental principles of IV-to-PO switching, which are founded on bioequivalence assessment, clinical stability criteria, and pharmacokinetic considerations remain applicable across different antimicrobial classes.

### Healthcare as complex systems

Healthcare delivery is a complex system consisting of inter-related elements with a common purpose.^108,109^ Systems operate at multiple levels including micro (individual clinician-patient interactions), meso (ward or departmental processes), and macro (institutional policies and national guidelines). IV-to-PO switch interventions that targeted single system levels or isolated components achieved modest, often unsustainable improvements, whilst multi-component interventions that addressed multiple system levels simultaneously achieved better outcomes; this is exemplified by the work of Buyle, Ciarkowski and van Niekerk, whose multi-component approaches were reported to be successful.^28,29,60^ Interventions that potentially did not account for cognitive workload, competing priorities, information flow, and decision-making workflows at the point of care demonstrated limited success regardless of evidence quality or guideline comprehensiveness. Conversely, interventions that takes into account other system factors, for example, those that distribute cognitive load across multidisciplinary teams at the precise moment of prescribing, may be more likely to achieve better outcomes. Studies that combined pharmacist expertise with electronic reminders achieved better outcomes to either element used independently in isolation.^35^ This outcome suggests that system-level interventions should be designed, implemented, and evaluated as integrated wholes rather than as collections of independent elements.

### Education based limitations

None of the studies reported utilized education as their sole intervention; all seven educational interventions were combined with other strategies. From a systems perspective, this finding may reflect that education alone is limited to the micro-level (individual clinician knowledge), without addressing meso-level processes (workflow, team communication) or macro-level structures (institutional policies and resource allocation). Education alone cannot overcome system-level barriers such as competing priorities or inadequate staffing. In the case of competing priorities, the wider literature indicates that resident doctors frequently face competing priorities that may lead them to forego timely intravenous (IV) to oral antibiotic switches in favour of tasks such as writing discharge letters and tending to acutely ill patients.^110^ Work conducted by Arnold and colleagues further supports the notion that the impact of educational interventions on outcomes related to the rate of IV-to-PO antibiotic switching is not sustained over time. In an ambulatory setting. Arnold *et al*. found that after implementing a single educational intervention, progressive decay in antibiotic switching practices occurred over time.^83^ Three mechanisms drove deterioration: staff turnover (40% of clinicians untrained within 2 years), competing clinical priorities, and absence of performance feedback. Only continuous reinforcement, such as quarterly refreshers, new hire training, email reminders with performance data, and dashboard integration were able stabilize switching rates at 15% points above baseline at 36 months (*P* < 0.001), preserving 83% of initial gains. This may suggest that at best, educational interventions when used in isolation are only capable of maintaining equilibrium rather than achieving progression in antimicrobial stewardship outcomes. For educational interventions to be successful, they must be combined with meso- and macro-level processes. This suggestion is exemplified by the work conducted by Ciarkowski *et al*. who found that whilst educating clinicians about IV-to-PO switching principles served as a necessary foundation in Phase I, combining clinician education with CDSS and active antimicrobial stewardship (AMS) team involvement for real-time consultation and intervention achieved maximal impact.

### Pharmacist-led interventions: expertise and interprofessional collaboration

Arguably, the limitations of education-based approaches and the documented competing priorities facing resident doctors support the integration of pharmacists into IV-to-PO switching decision-making. From a systems perspective, pharmacist-led interventions represent meso-level administrative controls that redistribute work across the healthcare team rather than relying on individual physician behaviour change. In the studies that evaluated pharmacist-led interventions and multidisciplinary teams, these studies demonstrated improved patient outcomes with regards to reduction in length of stay (LOS), a reduction in the duration of IV antibiotic therapy, and cost-savings compared to physician-only educational approaches. Pharmacists possess core training in pharmacokinetics, pharmacodynamics, and bioavailability, all of which are fundamental to identifying optimal antibiotic administration routes. This pharmaceutical expertise enables systematic evaluation of IV-to-PO switching criteria, including oral absorption characteristics and bioequivalence considerations. Additionally, some pharmacists have specialist training in antimicrobial stewardship and infectious diseases, providing enhanced capability for complex antimicrobial prescribing decisions. Whilst many pharmacists have competing priorities, such as discharge planning, medication reconciliation and clinical validation of medication, this interprofessional working model allows clinicians to work synergistically.

Interprofessional working has the potential to address multiple barriers simultaneously, in that it distributes cognitive workload across complementary expertise and creates peer accountability through collaborative review. In this respect, interprofessional working redesigns the system in which pharmacists and resident doctors work, rather than attempting to change individual behaviour within a potentially constrained system. Whilst pharmacists also face competing priorities including discharge planning, medication reconciliation, and clinical validation activities, interprofessional working models allow clinicians to function synergistically rather than duplicating efforts.

Van Schooneveld and colleague’s pharmacist-led antibiotic time-outs achieved 73.2% compliance not through education but through administrative control^59^: scheduled reviews with predetermined criteria applied by dedicated personnel with explicit authority to recommend changes. This represents a systems-level intervention operating at a meso-level, rather than individual-level education. The time-out structure creates a system design element that prevents progression until specific conditions are met. By scheduling regular reviews and designated responsibility, the intervention removes reliance on individual clinician memory or motivation to conduct an IV-to-PO switch. The scheduled nature of these ‘antibiotic time outs’ also potentially addresses the competing priorities problem: time-outs occur at predetermined intervals regardless of other demands, ensuring switching decisions receive attention within finite cognitive resources.

The evidence supports the notion that pharmacist-led interventions are effective in achieving IV-to-PO switches. Conversely, the apparent need for continuous pharmacist input may raise questions about whether the impact of pharmacist involvement lasts over prolonged periods of time. For example, Van Niekerk *et al*. reported that switching rates declined from 43.9% during active pharmacist involvement to 20.8% at three months post-intervention, approaching baseline levels. This may illustrates the resource-dependency of pharmacist-led models.^29^ The requirement for continuous dedicated pharmacist time, seven-day weekly coverage, and ongoing institutional prioritization may mean that pharmacist interventions, whilst more effective than education alone, remain vulnerable to resource reallocation and competing institutional priorities. These findings raise questions about optimal resource allocation at the macro-level. Specifically, healthcare systems must consider whether financial investment is better directed towards training pharmacists to work autonomously in initiating IV-to-PO switches versus employing pharmacists who require physician approval for all recommendations.

### Computerized decision support systems and digital transformation

The increasing adoption of electronic prescribing systems may alter the landscape for antimicrobial stewardship interventions. The current 5-year national plan for antimicrobial resistance (AMR) in the United Kingdom states that the next 5 years are crucial for ensuring AMR is considered an important part of digital transformation and that there is a need to adopt digital technologies that enhance AMS practices.^111^ Whilst healthcare environments may operate as hybrid paper-electronic systems during transitional periods, passive interventions that demonstrated some efficacy in paper-based systems, such as posters on walls, printed guidelines in case notes or educational pocket cards may continue to act as aide memoires alongside emerging digital interventions. Studies evaluating guideline implementation in this review predominantly examined paper-based passive dissemination or integration into physical medication charts; the extent to which these findings apply to fully electronic prescribing environments remains uncertain and warrants further investigation.

Clinical decision support systems (CDSS) are digital tools that can support clinicians to make more equitable, evidence-based decisions. The ‘Five Rights’ of CDS (the right information, to the right person, in the right intervention format, through the right channel, at the right time in the workflow offer guidance to developers to create successful systems.^112^ In this respect, our findings highlight that studies with CDSS that aligned with these five principles were successful, whereas studies that appeared to deviate from the CDS principles were less successful. For example, the Fischer *et al*. study demonstrated effective CDSS design by embedding prompts at the point of prescribing,^68^ the precise moment when prescribers accessed the system to make antibiotic-related decisions. The 11.1% reduction in IV defined daily doses reflected the property of well-designed human-technology systems and met the ‘right time’ criterion. Similarly, Xu and colleagues reported that combining computerized reminders with pharmacist interventions improved efficacy by delivering alerts to the ‘right person’ with antimicrobial expertise to make better prescribing choices.^35^ Electronic health record systems create opportunities for interventions aligned with CDS Five Rights principles. Future work should focus on developing CDSS interventions that are aligned with the CDS Five Rights principles.

The wider literature base comments on alert fatigue, a limitation of electronic-based systems. Clinicians override the vast majority of prescribing alerts, with override rates frequently exceeding 90%.^113^ Alert acceptance decreased by 30% for each additional alert per encounter,^114^ and other studies have emphasized that clinicians’ negative perceptions regarding the clinical relevance of frequent alerts contribute to low acceptance rates.^115^ Such studies suggest that both the absolute number of alerts and the frequency of repeated alerts synergistically reduce the clinical uptake of CDSS recommendations.^116,117^ Successful CDSS implementation requires not merely digitizing existing paper-based guidelines, but redesigning decision support aligned with human factors principles. This includes tiered alerts based on genuine clinical severity, elimination of repetitive low-value alerts, suppression mechanisms when previous clinical decisions have addressed concerns, and integration with workflow at precise decision points rather than interrupting unrelated activities

Effective electronic interventions aligned with NHS 5-year plan objectives will likely require hybrid approaches combining CDSS elements (alerts identifying patients meeting switching criteria) with facilitated pharmacist review (alerts directing pharmacist attention to specific patients) and streamlined communication pathways (enabling pharmacists to document recommendations directly in prescribing interfaces). Such integrated systems leverage technology’s strengths in data monitoring, pattern recognition, and timely alerting whilst preserving human clinical judgment and interprofessional collaboration for complex decision-making. This represents optimal human-technology system design: automating routine data processing and monitoring whilst reserving human cognition for nuanced clinical judgment that requires contextual understanding.

### Clinician attitudes and respiratory-specific concerns

One notable barrier for physicians making IV-to-PO switches was clinician attitude. The current literature indicates that respiratory clinicians often modify traditional antimicrobial practices when managing pleural infections due to concerns regarding the adequacy of oral antibiotic penetration into pleural tissue.^118–120^ Despite physician concerns of oral efficacy, the use of oral antibiotics can be as effective as IV regimens.^121–123^ These findings are significant because they challenge the longstanding instinct to rely solely on IV therapy when therapeutic levels in difficult-to-penetrate tissues like the pleura are of concern.

### Implications for practice and future research

Current guidelines for IV to PO conversion tend to be generalized and primarily based on systemic parameters such as fever resolution and overall clinical improvement, without consideration of respiratory-specific factors.^124^ Clinical parameters such as respiratory rate, oxygen saturation, and the presence of chronic pulmonary conditions are rarely incorporated into these guidelines. Yaakoubi and colleagues, for example, have highlighted that persistent respiratory distress, such as hypoxaemia, may continue even when systemic signs of infection have improved.^125^ This suggests that standardized protocols for transitioning to oral antibiotics may inadequately address the dynamic and nuanced respiratory status of patients, potentially leading to suboptimal therapeutic decisions. From a systems perspective, protocols or guidelines that do not account for context-specific clinical factors have the potential to create misalignment between ‘standardized’ protocols and actual clinical practice. In this respect, there is the potential for a larger disparity between ‘work-as-imagined’ versus ‘work-as-done’ which may undermine antimicrobial stewardship objectives.

The creation of decision aids tailored to meet the needs of physicians treating respiratory conditions may address this gap. However, the design and implementation of such decision aids must account for the heterogeneity in health information technology infrastructure across healthcare settings. The transition from paper-based to fully electronic prescribing systems will likely take some time, with potential variation between institutions and healthcare systems; this is supported by the work of Garfield and colleagues, who reported that only 13% of 101 English trusts had electronic prescribing systems in all surgical and medical wards, with many hospitals using electronic prescribing in certain clinical areas only.^126^ During this transitional period, effective interventions must function across hybrid environments where both paper charts and electronic systems coexist. This necessitates a dual approach: developing electronic decision support tools optimized for digital prescribing interfaces while simultaneously maintaining accessible paper-based decision aids (such as integrated chart prompts or bedside reference cards) for settings where electronic systems are unavailable or incomplete.

The evidence from this review, which predominantly evaluated paper-based interventions, remains relevant for such hybrid environments and for healthcare settings that have not yet implemented comprehensive electronic prescribing systems gap.

Future research should explicitly adopt systems thinking and human factors approaches when designing, implementing, and evaluating IV-to-PO switching interventions. Specific priorities include determining differences of respiratory-specific versus generalized switching criteria and designing effective CDSS that minimizes alert fatigue and applying human factors principles to information display, timing, and integration. This work could take the form of identifying optimal models for pharmacist integration to achieve more frequent IV-to-PO antibiotic switches, or investigating and designing electronic prescribing interventions that are reflective of physicians perceived wants and needs to maximize IV-to-PO switching in pneumonia management to make way for better clinical practices. While our findings suggest valuable intervention components, particularly clinical decision support systems aligned with the global shift towards electronic prescribing, enhanced pharmacist involvement in antimicrobial optimization, and workflow-integrated protocols, the optimal combination and implementation strategy likely varies by institutional capacity, baseline infrastructure, and available resources.

### Limitations

Some studies lacked adequate control for temporal trends and seasonal variation in pneumonia severity. Additionally, multiple studies made use of multiple interventions simultaneously; this systematic review makes it clear that these interventions are effective, but it remains challenging to determine whether specific interventions are more effective than others. Secondly, we note that the predominance of adult populations compared to paediatric populations limits generalisability to paediatric settings. This imbalance likely reflects several factors: differing clinical practice patterns between adult and paediatric populations, more stringent ethical requirements for paediatric research including parental consent, and distinct pharmacokinetic considerations affecting IV-to-PO switching in children including weight-based dosing, formulation availability, and palatability concerns. The limited paediatric evidence base highlights an important research gap, as intervention strategies effective in adults may require substantial adaptation for paediatric populations. No studies evaluated whether interventions were able to achieve the same clinical and economic outcomes beyond 12–24 months; most studies reported only immediate post-intervention outcomes. The documented decay phenomenon suggests many interventions demonstrate short-term effectiveness, which may achieve minimal sustained impact. We also recognize the heterogeneity of outcome measures reported, which limits the ability to conduct one to one comparison and conduct meta-analysis.

## Conclusion

This systematic review identified that IV-to-PO antibiotic switch interventions are effective when used in the management of pneumonia, but the optimal design to achieve efficacy remains unclear. The review findings suggest that intervention effectiveness depends less on guideline quality or evidence strength than on how interventions address the complex interplay of individual, team, technological, and organizational factors within healthcare systems.

Multi-component interventions combining elements at different system levels achieved better outcomes compared to single-component approaches. Studies directly comparing intervention intensities demonstrated stepwise improvements as components were added, reflecting synergistic interactions between different system elements. Pharmacist-led interventions consistently improved IV therapy duration, length of stay, and costs by redistributing specialized tasks to appropriately trained personnel. Computerized decision support systems offer potential for sustainable intervention as engineering controls operating independently of continuous personnel oversight. However, effectiveness depends critically on its design: providing information at the right time, to the right person, in formats aligned with cognitive capabilities and workflow. Poor CDSS design generates alert fatigue and systematic override behaviour, demonstrating that technology alone cannot overcome poorly designed work systems.

We suggest that healthcare systems should consider interventions that restructure work processes and distribute responsibility rather than relying primarily on individual clinician behaviour change. This includes using Human Factors and Ergonomics to inform intervention design and its implementation e.g. in integrating pharmacists into switching decision-making, procuring, designing and implementing electronic decision support, and establishing macro-level policies that support sustained resource allocation to antimicrobial stewardship infrastructure. By recognizing IV-to-PO switching as a system-level challenge requiring coordinated modification across multiple levels rather than an individual knowledge deficit requiring education, healthcare organizations can work towards the sustained improvements in antimicrobial stewardship that patient safety and antimicrobial resistance mitigation require.

## Supplementary Material

dlag065_Supplementary_Data

## Supplementary data

Tables S1, S2 and S3 are available in supplementary data.
