# Supplementary material for: The effectiveness of intravenous (IV) to oral (PO) antibiotic switch (IVOS) interventions in managing community- and hospital-acquired pneumonia—a systematic review
Source: JAC Antimicrob Resist. 2026 May 12;8(3):dlag065. doi: 10.1093/jacamr/dlag065 (PMC13160672; doi:10.1093/jacamr/dlag065)
Supplement: dlag065_Supplementary_Data [file dlag065_supplementary_data.zip › MMAT S3.pdf]

				SCREENING QUESTIONS		1. QUALITATIVE STUDIES					2. RANDOMIZED CONTROLLED TRIALS					3. NON-RANDOMIZED STUDIES					4. QUANTITATIVE DESCRIPTIVE STUDIES					5. MIXED METHODS STUDIES				
RefID	First author	Year	Citation	S1. Are there clear research questions?	S2. Do the collected data allow to address the research questions?	1.1. Is the qualitative approach appropriate to answer the research question?	1.2. Are the qualitative data collection methods adequate to address the research question?	1.3. Are the findings adequately derived from the data?	1.4. Is the interpretation of results sufficiently substantiated by data?	1.5. Is there coherence between qualitative data sources, collection, analysis and interpretation?	2.1. Is randomization appropriately performed?	2.2. Are the groups comparable at baseline?	2.3. Are there complete outcome data?	2.4. Are outcome assessors blinded to the intervention provided?	2.5 Did the participants adhere to the assigned intervention?	3.1. Are the participants representative of the target population?	3.2. Are measurements appropriate regarding both the outcome and intervention (or exposure)?	3.3. Are there complete outcome data?	3.4. Are the confounders accounted for in the design and analysis?	3.5. During the study period, is the intervention administered (or exposure occurred) as intended?	4.1. Is the sampling strategy relevant to address the research question?	4.2. Is the sample representative of the target population?	4.3. Are the measurements appropriate?	4.4. Is the risk of nonresponse bias low?	4.5. Is the statistical analysis appropriate to answer the research question?	5.1. Is there an adequate rationale for using a mixed methods design to address the research question?	5.2. Are the different components of the study effectively integrated to answer the research question?	5.3. Are the outputs of the integration of qualitative and quantitative components adequately interpreted?	5.4. Are divergences and inconsistencies between quantitative and qualitative results adequately addressed?	5.5. Do the different components of the study adhere to the quality criteria of each tradition of the methods involved?
1	Grill	2011	Grill E, Weber A, Lohmann S, Vetter-Kerkhoff C, Strobl R, Jauch K-W. Effects of pharmaceutical counselling on antimicrobial use in surgical wards: Intervention study with historical control group. Pharmacoepidemiology and Drug Safety. 2011;20(7):739-46.	Yes	Yes											Yes	Yes	Yes	Yes	Can't tell										
2	Oosterheert	2006	Oosterheert J, Bonten M, Schneider M, Buskens E, Lammers J, Hustinx W, et al. Effectiveness of early switch from intravenous to oral antibiotics in severe community acquired pneumonia: multicentre randomised trial. BMJ-BRITISH MEDICAL JOURNAL. 2006;333(7580):1193-5.	Yes	Yes						Yes	Yes	Yes	Yes	Yes															
3	Buyle	2012	Buyle FM, Metz-Gercek S, Mechtler R, Kern WV, Robays H, Vogelaers D, et al. Prospective multicentre feasibility study of a quality of care indicator for intravenous to oral switch therapy with highly bioavailable antibiotics. Journal of Antimicrobial Chemotherapy. 2012;67(8):2043-6.	Yes	Yes											Yes	Yes	Yes	Yes	Can't tell										
4	van Niekerk	2011	van Niekerk AC, Venter DJL, Boschmans S-A. Implementation of intravenous to oral antibiotic switch therapy guidelines in the general medical wards of a tertiary-level hospital in South Africa. Journal of Antimicrobial Chemotherapy. 2011;67(3):756-62.	Yes	Yes											Yes	Yes	Yes	Can't tell	Yes										
5	Emerson	2008	Emerson CR, Antonopoulos MS, Marzella N, Grossman SS. Economic impact of implementing pneumonia treatment guidelines for intravenous to oral conversion. Hospital Pharmacy. 2008;43(11):886-94.	Yes	Yes											Yes	Yes	Yes	Can't tell	Yes										
6	Yaqub	2005	Yaqub A, Khan Z. Comparison of early intravenous to oral switch amoxicillin/clavulanate with parenteral ceftriaxone in treatment of hospitalized patients with community acquires pneumonia. 2005;21(3):259-66.	Yes	Yes						Yes	Yes	Yes	No	Yes															
7	In-iw	2015	In-iw S, Winijkul G, Sonjaipanich S, Manaboriboon B. Comparison between the Efficacy of Switch Therapy and Conventional Therapy in Pediatric Community-Acquired	Yes	Yes						Yes	Yes	Yes	Can't tell	Yes															
8	Castro-Guardiola	2001	Castro-Guardiola A, Viejo-Rodríguez A, Soler-Simon S, Armengou-Arxé A, Bisbe-Company V, Peñarroja-Matutano G, et al. Efficacy and safety of oral and early-switch therapy for community-acquired pneumonia: a randomized controlled trial. 2001;111(5):367-74.	Yes	Yes						Yes	Yes	Yes	No	Yes															
9	Van der Eerden	2004	Van der Eerden MM, de Graaff CS, Vlaspolder F, Bronsveld W, Jansen HM, Boersma WG. Evaluation of an algorithm for switching from IV to PO therapy in clinical practice in patients with community-acquired pneumonia. Clinical Therapeutics. 2004;26(2):294-303.	Yes	Yes											Yes	Yes	Yes	Can't tell	Yes										
10	Al-Eidan	1999	Al-Eidan FA, McElnay JC, Scott MG, Kearney MP, Troughton KEU, Jenkins J. Sequential antimicrobial therapy: Treatment of severe lower respiratory tract infections in children. Journal of Antimicrobial Chemotherapy. 1999;44(5):709-15.	Yes	Yes											Yes	Yes	Can't tell	Can't tell	Can't tell										
11	Hendrickson	1995	Hendrickson JR, North DS. Pharmacoeconomic Benefit of Antibiotic Step-Down Therapy: Converting Patents from Intravenous Ceftriaxone to Oral Cefpodoxime Proxettl. Annals of Pharmacotherapy. 1995;29(6):561-5.	Yes	Yes											Yes	Yes	Can't tell	Can't tell	Can't tell										
12	Xu	2021	Xu S, Wang X, Song Z, Han F, Zhang C. Impact and barriers of a pharmacist-led practice with computerized reminders on intravenous to oral antibiotic conversion for community-acquired pneumonia inpatients. J Clin Pharm Ther. 2021 Aug;46(4):1055-1061. doi: 10.1111/jcpt.13397. Epub 2021 Jun 7. PMID: 34101230.	Yes	Yes											Yes	Yes	Yes	Can't tell	Yes										
13	Frei	2006	Frei CR, Restrepo MI, Mortensen EM, Burgess DS. Impact of Guideline-Concordant Empiric Antibiotic Therapy in Community-Acquired Pneumonia. The American Journal of Medicine. 2006;119(10):865-71.	Yes	Yes											Yes	Yes	Can't tell	Can't tell	Can't tell										

14	Mazzola	2005	Mazzola JL, Schaefer OP, DeBellis RJ, Volturo GA. Evaluation of antibiotic usage with a local community-acquired pneumonia guideline. Infectious Diseases in Clinical Practice. 2005;13(2):60-4.	Yes	Yes						Yes	Yes	Can't tell	Can't tell	Can't tell					
15	Shrayteh	2014	Shrayteh Z, Rahal M, Malaeb D, Shrayteh ZM, Rahal MK, Malaeb DN. Practice of switch from intravenous to oral antibiotics. SPRINGERPLUS. 2014;3.	Yes	Yes						Yes	Yes	Can't tell	Can't tell	Can't tell					
16	Christensen	2019	Eric W Christensen, Alicen Burns Spaulding, William F Pomputius, Steven P Grapentine, Effects of Hospital Practice Patterns for Antibiotic Administration for Pneumonia on Hospital Lengths of Stay and Costs, Journal of the Pediatric Infectious Diseases Society, Volume 8, Issue 2, June 2019, Pages 115–121, https://doi.org/10.1093/jpids/piy003	Yes	Yes						Yes	Yes	Yes	Can't tell	Yes					
17	Fok	2002	MC Fok, Z Kanji, R Mainra, M Boldt. Characterizing and developing strategies for the treatment of communityacquired pneumonia at a community hospital. Can Respir J 2002;9(4):247-252.	Yes	Yes						Yes	Yes	Can't tell	Can't tell	Yes					
18	Buyle	2010	Buyle F, Vogelaers D, Peleman R, Maele G, Robays H. Implementation of guidelines for sequential therapy with fluoroquinolones in a Belgian hospital. Pharmacy World & Science. 2010;32(3):404-10.	Yes	Yes						Yes	Yes	Yes	Yes	Can't tell					
19	Hagaman	2005	Hagaman JT, Yurkowski P, Trott A, Rouan GW. Getting physicians to make "the switch": the role of clinical guidelines in the management of community-acquired pneumonia. American journal of medical quality. 2005;20(1):15-21.	Yes	Yes						Yes	Yes	Yes	Yes	Yes					
20	Lee	2006	Lee RWW, Lindstrom ST. Early switch to oral antibiotics and early discharge guidelines in the management of community-acquired pneumonia. Respirology. 2006;12(1):111-6.	Yes	Yes						Yes	Yes	Yes	Can't tell	Yes					
21	Mouwen	2020	Mouwen A, Dijkstra J, Jong E, Buijtels P, Pasker-De Jong P, Nagtegaal J, et al. Early switching of antibiotic therapy from intravenous to oral using a combination of education, pocket-sized cards and switch advice: A practical intervention resulting in reduced length of hospital stay. INTERNATIONAL JOURNAL OF ANTIMICROBIAL AGENTS. 2020;55(1).	Yes	Yes											Yes	Yes	Yes	Yes	Yes
22	Lesprit	2010	Lesprit P, Landelle C, Girou E, Brun-Buisson C. Reassessment of intravenous antibiotic therapy using a reminder or direct counselling. Journal of Antimicrobial Chemotherapy. 2010;65(4):789-95.	Yes	Yes						Yes	Yes	Can't tell	Can't tell	Can't tell					
23	Ahmed	2018	Ahmed SA, Kumar A, Sethi P, Kapil A, Pandey RM, Wig N. Effectiveness of education and antibiotic control programme at All India Institute of Medical Sciences, New Delhi. Natl Med J India. 2018 Sep-Oct;31(5):262-267. doi: 10.4103/0970-258X.261176. PMID: 31267989.	Yes	Yes											Yes	Yes	Yes	Yes	Yes
24	Dunn	2011	Dunn K, O'Reilly A, Silke B, Rogers T, Bergin C, Dunn K, et al. Implementing a pharmacist-led sequential antimicrobial therapy strategy: a controlled before-and-after study. INTERNATIONAL JOURNAL OF CLINICAL PHARMACY. 2011;33(2):208-14.	Yes	Yes						Yes	Yes	Yes	Yes	Yes					
25	Maripuu	2014	Maripuu H, Aldeyab M, Kearney M, McElnay J, Conlon G, Magee F, et al. An audit of antimicrobial treatment of lower respiratory and urinary tract infections in a hospital setting. EUROPEAN JOURNAL OF HOSPITAL PHARMACY. 2014;21(3):139-44.	Yes	Yes						Yes	Yes	Yes	Can't tell	Yes					
26	Eron	2001	Eron L, Passos S, Eron L, Passos S. Early discharge of infected patients through appropriate antibiotic use. ARCHIVES OF INTERNAL MEDICINE. 2001;161(1):61-5.	Yes	Yes						Yes	Yes	Can't tell	Yes	Yes					
27	Omidvari	1998	Omidvari K, de Boisblanc B, Karam G, Nelson S, Haponik E, Summer W. Early transition to oral antibiotic therapy for community-acquired pneumonia: duration of therapy, clinical outcomes, and cost analysis. 1998:92(8):1032-9.	Yes	Yes	Yes	Yes	Yes	No	Yes										
28	DiDiodato	2016	DiDiodato, G. et al. (2016) 'Evaluating the impact of an antimicrobial stewardship program on the length of stay of immune-competent adult patients admitted to a hospital ward with a diagnosis of community-acquired pneumonia: A quasi-experimental study', American Journal of Infection Control, 44(5), pp. e73–e79. Available at: https://doi.org/10.1016/j.ajic.2015.12.026.	Yes	Yes											Yes	Yes	Yes	Yes	Yes
29	Jarab	2018	Jarab A, Mukattash T, Nusairat B, Shawaqfeh M, Abu Farha R, Jarab AS, et al. Patterns of antibiotic use and administration in hospitalized patients in Jordan. SAUDI PHARMACEUTICAL JOURNAL. 2018;26(6):764-70.	Yes	Yes											Yes	Yes	Yes	Yes	Yes

30	Walker	2023	Walker R, Pallotta A, Schulte R, Tereshchenko L, Criswell V, Deshpande A, et al. Acceptance of Pharmacist-Led Stewardship Recommendations for Patients with Community-Acquired Pneumonia. 2023:10:S1139-S40.	Yes	Yes											Yes	Yes	Yes	Yes	Yes
31	Clark	2000	Clark LC, Davis CW. Experiences at a large teaching hospital with levofloxacin for the treatment of community-acquired pneumonia. American Journal of Health-System Pharmacy. 2000;57(suppl_3):S10-S3.	Yes	Yes						Yes	Yes	Can't tell	Can't tell	Can't tell					
32	Schouten	2007	Schouten J, Hulscher M, Trap-Liefers J, Akkermans R, Kullberg B, Grol R, et al. Tailored interventions to improve antibiotic use for lower respiratory tract infections in hospitals: A cluster-randomized, controlled trial. CLINICAL INFECTIOUS DISEASES. 2007;44(7):931-41.	Yes	Yes	Yes	Yes	Yes	Yes	Yes										
33	Zaal	2020	Zaal RJ, Den Haak EW, Andrinopoulou ER, Van Gelder T, Vulto AG, Van Den Bemt PMLA. Physicians' acceptance of pharmacists' interventions in daily hospital practice. International Journal of Clinical Pharmacy. 2020;42(1):141-9.	Yes	Yes						Yes	Yes	Yes	Can't tell	Yes					
34	Ramirez	1999	Ramirez JA, Vargas S, Ritter GW, Brier ME, Wright A, Smith S, et al. Early Switch From Intravenous to Oral Antibiotics and Early Hospital Discharge. Archives of Internal Medicine. 1999;159(20):2449.	Yes	Yes						Yes	Yes	Can't tell	Can't tell	Can't tell					
35	Fine	2003	Fine MJ, Stone RA, Lave JR, Hough LJ, Obrosky DS, Mor MK, et al. Implementation of an evidence-based guideline to reduce duration of intravenous antibiotic therapy and length of stay for patients hospitalized with community-acquired pneumonia: a randomized controlled trial. The American Journal of Medicine. 2003;115(5):343-51.	Yes	Yes	Yes	Yes	Yes	Can't tell	Can't tell										
36	Carratela	2012	Carratalà J, Garcia-Vidal C, Ortega L, et al. Effect of a 3-step critical pathway to reduce duration of intravenous antibiotic therapy and length of stay in community-acquired pneumonia: a randomized controlled trial: a 3-step critical pathway for CAP. Arch Intern Med 2012;172:922–8.	Yes	Yes	Yes	Yes	Yes	No	Yes										
37	Feagan	2001	Feagan BG. A controlled trial of a critical pathway for treating community-acquired pneumonia: the CAPITAL study. Community-Acquired Pneumonia Intervention Trial Assessing Levofloxacin. Pharmacotherapy. 2001 Jul;21(7 Pt 2):89S-94S. doi: 10.1592/phco.21.10.89s.34535. PMID: 11446524.	Yes	Yes	Yes	Yes	Yes	Yes	Yes										
38	Di Giammarino	2005	Di Giammarino L, Bihl F, Bissig M, Bernasconi B, Cerny A, Bernasconi E. Evaluation of prescription practices of antibiotics in a medium-sized Swiss hospital. Swiss Med Wkly. 2005 Dec 10;135(47-48):710-4. doi: 10.4414/smw.2005.11174. PMID: 16511707.	Yes	Yes						Yes	Yes	Yes	Can't tell	Yes					
39	Yen	2012	Yen Y, Chen H, Wuan-Jin L, Lin Y, Shen W, Cheng K, et al. Clinical and economic impact of a pharmacist-managed i.v.-to-p.o. conversion service for levofloxacin in Taiwan. INTERNATIONAL JOURNAL OF CLINICAL PHARMACOLOGY AND THERAPEUTICS. 2012;50(2):136-41.	Yes	Yes	Yes					Yes	Yes	Yes	Can't tell	Yes					
40	Laing	1998	Laing R. The effect of intravenous-to-oral switch guidelines on the use of parenteral antimicrobials in medical wards. Journal of Antimicrobial Chemotherapy. 1998;42(1):107-11.	Yes	Yes											Yes	Yes	Yes	Yes	Yes
41	Van Der Bergh	2020	Van Den Bergh D, Messina AP, Goff DA, Van Jaarsveld A, Coetzee R, De Wet Y, et al. A pharmacist-led prospective antibiotic stewardship intervention improves compliance to community-acquired pneumonia guidelines in 39 public and private hospitals across South Africa. International Journal of Antimicrobial Agents. 2020;56(6):106189.	Yes	Yes	Yes	Yes	Yes	Can't tell	Can't tell										
42	Ciarkowski	2020	Ciarkowski, C.E. et al. (2020) 'A Pathway for Community-Acquired Pneumonia With Rapid Conversion to Oral Therapy Improves Health Care Value', Open Forum Infectious Diseases, 7(11). Available at: https://doi.org/10.1093/ofid/ofaa497.	Yes	Yes											Yes	Yes	Yes	Can't tell	Can't tell
43	Nguyen	2023	Nguyen TNT, Bui QTH, Tran VT, Tran NQ, Nguyen NTY, Nguyen HT, et al. Impact of clinical pharmacist-led interventions on switching from intravenous-to-oral antibiotics in patients with infectious diseases at a Vietnamese hospital. Trop Med Int Health. 2023;28(8):612-9.	Yes	Yes						Yes	Yes	Yes	Can't tell	Yes					
44	Anusha	2021	Anusha B, Shanmugam P, Anil Kumar T, Vasista S, Stephy C, Subeesh V. Early conversion of intravenous to oral antibiotic therapy in uncomplicated urinary and respiratory tract infection. Drugs & Therapy Perspectives. 2021;37(4):181-6.	Yes	Yes						Yes	Yes	Yes	Can't tell	Yes					

45	Sze	2018	Sze WT, Kong MC. Impact of printed antimicrobial stewardship recommendations on early intravenous to oral antibiotics switch practice in district hospitals. Pharmacy Practice. 2018;16(2).	Yes	Yes	Yes	Yes	Can't tell	Can't tell	Can't tell
46	Tejaswini	2018	Tejaswini Y, Challa S, Nalla K, Gadde R, Pavani A, Neerisha V, et al. Practice of Intravenous to Oral Conversion of Antibiotics and its Influence on Length of Stay at a Tertiary Care Hospital: A Prospective Study. JOURNAL OF CLINICAL AND DIAGNOSTIC RESEARCH. 2018;12(3):FC01-FC4.	Yes	Yes	Yes	Yes	Yes	Can't tell	Yes
47	Engel	2014	Engel MF, Bruns AHW, Hulscher MEJL, Gaillard CAJM, Sankatsing SUC, Teding van Berkhout F, et al. A tailored implementation strategy to reduce the duration of intravenous antibiotic treatment in community-acquired pneumonia: a controlled before-and-after study. European Journal of Clinical Microbiology & Infectious Diseases. 2014;33(11):1897-908.	Yes	Yes	Yes	Yes	Yes	Yes	Yes
48	Fésüs	2024	Fésüs A, Baluku P, Sipos É, Somodi S, Berczi-Kun E, Lekli I, Bácskay I, Benkő R, Vaskó A. The effect of the antibiotic stewardship program (ASP) on community-acquired pneumonia (CAP): a before-after study. Front Pharmacol. 2024 Aug 6;15:1406960. doi: 10.3389/fphar.2024.1406960. PMID: 39166111; PMCID: PMC11333452.	Yes	Yes	Yes	Yes	Yes	Can't tell	Yes
49	Kuti	2002	Kuti JL, Le TN, Nightingale CH, Nicolau DP, Quintiliani R. Pharmacoeconomics of a pharmacist-managed program for automatically converting levofloxacin route from i.v. to oral. American Journal of Health-System Pharmacy. 2002;59(22):2209-15.	Yes	Yes	Yes	Yes	Yes	Can't tell	Yes
50	Fischer	2003	Fischer MA, Solomon DH, Teich JM, Avorn J. Conversion from Intravenous to Oral Medications: Assessment of a Computerized Intervention for Hospitalized Patients. Archives of Internal Medicine. 2003;163(21):2585-9.	Yes	Yes	Yes	Yes	Yes	Can't tell	Yes
51	Golali	2019	Golali E, Sistanizad M, Salamzadeh J, Haghighi M, Solooki M. Antibiotic prescribing trends before and after implementation of an audit and feedback program in internal ward of a tertiary hospital in tehran. Iranian Journal of Pharmaceutical Research. 2019;18(4):2136-43.	Yes	Yes	Yes	Yes	Yes	No	Yes
52	Kawamura	2017	Kawamura, M. et al. (2017) 'Effectiveness of an early switch from intravenous to oral antimicrobial therapy for lower respiratory tract infection in patients with severe motor intellectual disabilities', Journal of Infection and Chemotherapy, 24(1), pp. 40–44. Available at: https://doi.org/10.1016/j.jiac.2017.08.017.	Yes	Yes	Yes	Yes	Yes	Can't tell	Yes
53	Shindo	2008	Shindo Y, Sato S, Maruyama E, Ohashi T, Ogawa M, Imaizumi K, et al. Implication of Clinical Pathway Care for Community-Acquired Pneumonia in a Community Hospital: Early Switch from an Intravenous .BETA.-lactam Plus a Macrolide to an Oral Respiratory Fluoroquinolone. Internal Medicine. 2008;47(21):1865-74.	Yes	Yes	Yes	Yes	Yes	Can't tell	Yes
54	Melo Rodrigues	2013	Melo Rodrigues R, Fontes AMS, César Mantese O, Souza Martins R, Tanús Jorge M. Impact of an intervention in the use of sequential antibiotic therapy in a Brazilian university hospital. Revista da Sociedade Brasileira de Medicina Tropical. 2013;46(1):50-4.	Yes	Yes	Yes	Yes	Yes	Can't tell	Yes
55	Sadeq	2021	Sadeq AA, Shamseddine JM, Babiker ZOE, Nsutebu EF, Moukarzel MB, Conway BR, et al. Impact of multidisciplinary team escalating approach on antibiotic stewardship in the United Arab Emirates. Antibiotics. 2021;10(11).	Yes	Yes	Yes	Yes	Yes	Can't tell	Yes
56	Peyrani	2013	Peyrani P, Ramirez J. What is the Association of Cardiovascular Events with Clinical Failure in Patients with Community-Acquired Pneumonia? Infectious Disease Clinics of North America. 2013;27(1):205-10.	Yes	Yes	Yes	Yes	Yes	Can't tell	Yes
57	Halley	2000	Halley HJ. Approaches to drug therapy, formulary, and pathway management in a large community hospital. American Journal of Health-System Pharmacy. 2000;57(suppl_3):S17-S21.	Yes	Yes	Yes	Yes	Yes	Can't tell	Yes
58	Davis	2005	Davis SL, Delgado G, McKinnon PS. Pharmacoeconomic Considerations Associated with the Use of Intravenous-to-Oral Moxifloxacin for Community-Acquired Pneumonia. Clinical Infectious Diseases. 2005;41:S136-S43.	Yes	Yes	Yes	Yes	Yes	Can't tell	Yes
59	Van Schooneveld	2020	Van Schooneveld TC, Rupp ME, Cavaleiri RJ, Lyden E, Rolek K. Cluster randomized trial of an antibiotic time-out led by a team-based pharmacist. Infection Control & Hospital Epidemiology. 2020;41(11):1266-1271. doi:10.1017/ice.2020.347	Yes	Yes	Yes	Yes	Yes	Can't tell	Yes
