# Supplementary material for: The effectiveness of intravenous (IV) to oral (PO) antibiotic switch (IVOS) interventions in managing community- and hospital-acquired pneumonia—a systematic review
Source: JAC Antimicrob Resist. 2026 May 12;8(3):dlag065. doi: 10.1093/jacamr/dlag065 (PMC13160672; doi:10.1093/jacamr/dlag065)
Supplement: dlag065_Supplementary_Data [file dlag065_supplementary_data.zip › Search Strategy S2.docx]

**Search Strategy – Supplementary Data 2**

**Cochrane Library**

| ID | Search |
| --- | --- |
| #1 | ("community acquired pneumonia"):ti,ab,kw (Word variations have been searched) |
| #2 | (CAP):ti,ab,kw (Word variations have been searched) |
| #3 | (HAP):ti,ab,kw |
| #4 | ("hospital acquired pneumonia"):ti,ab,kw (Word variations have been searched) |
| #5 | (IV-to-PO antibiotic transition):ti,ab,kw (Word variations have been searched) |
| #6 | (hospital):ti,ab,kw (Word variations have been searched) |
| #7 | (healthcare settings):ti,ab,kw (Word variations have been searched) |
| #8 | (decision aid):ti,ab,kw (Word variations have been searched) |
| #9 | (clinical decision support tool):ti,ab,kw (Word variations have been searched) |
| #10 | (clinical pathways):ti,ab,kw (Word variations have been searched) |
| #11 | (clinical guidelines):ti,ab,kw (Word variations have been searched) |
| #12 | (clinical outcomes):ti,ab,kw (Word variations have been searched) |
| #13 | (readmission):ti,ab,kw (Word variations have been searched) |
| #14 | (relapse):ti,ab,kw (Word variations have been searched) |
| #15 | (hospital discharge):ti,ab,kw (Word variations have been searched) |
| #16 | (intravenous to oral antibiotic transition):ti,ab,kw (Word variations have been searched) |
| #17 | (IVOS):ti,ab,kw (Word variations have been searched) |
| #18 | (intravenous to oral antibiotic switch):ti,ab,kw (Word variations have been searched) |
| #19 | (Clinical decision support systems):ti,ab,kw (Word variations have been searched) |
| #20 | (prescribing practices):ti,ab,kw (Word variations have been searched) |
| #21 | (recurrence):ti,ab,kw (Word variations have been searched) |
| #22 | (treatment failure):ti,ab,kw (Word variations have been searched) |
| #23 | (length of stay):ti,ab,kw (Word variations have been searched) |
| #24 | (clinician opinion):ti,ab,kw (Word variations have been searched) |
| #25 | (clinician engagement):ti,ab,kw (Word variations have been searched) |
| #26 | (clinician satisfaction):ti,ab,kw (Word variations have been searched) |
| #27 | #1 OR #2 OR #3 OR #4 |
| #28 | #6 OR #7 |
| #29 | #5 OR #16 OR #17 OR #18 |
| #30 | #8 OR #9 OR #10 OR #11 OR #19 OR #20 |
| #31 | #12 OR #13 OR #14 OR #15 OR #21 OR #22 OR #23 OR #24 OR #25 OR #26 |
| #32 | #27 AND #28 AND #29 AND #30 AND #31 |
| #33 | ((pneumonia OR "community-acquired pneumonia" OR "hospital-acquired pneumonia" OR "ventilator-associated pneumonia" OR "healthcare-associated pneumonia" OR "lower respiratory tract infection" OR LRTI OR RTI OR "chest infection")):ti,ab,kw AND (("intravenous to oral switch" OR "IV to PO switch" OR "oral step-down therapy" OR "sequential therapy" OR "switch therapy" OR "early switch" OR IVOS OR ("intravenous" AND "oral" AND antibiotics))):ti,ab,kw AND ((hospitalised OR hospitalized OR inpatient OR "acute care" OR "secondary care" OR "tertiary care" OR "community hospital")):ti,ab,kw AND (("length of stay" OR "duration of therapy" OR "clinical outcomes" OR "treatment failure" OR readmission OR "early discharge" OR "cost-effectiveness" OR "clinical stability" OR "therapeutic success")):ti,ab,kw (Word variations have been searched) |

**Web of Science**

TS=(pneumonia OR pneumonias OR pneumoniae OR "community-acquired pneumonia" OR CAP OR

"hospital-acquired pneumonia" OR HAP OR "ventilator-associated pneumonia" OR VAP OR

"healthcare-associated pneumonia" OR HCAP OR "lower respiratory tract infection" OR

LRTI OR "respiratory tract infection" OR RTI OR "chest infection")

AND

TS=("intravenous to oral switch" OR "IV to PO switch" OR "IV to PO transition" OR

"oral step-down therapy" OR "step-down therapy" OR "sequential therapy" OR

"switch therapy" OR "early switch" OR "early discharge" OR inos OR

"IV-to-oral conversion" OR "IV-PO conversion" OR

((intravenous OR "intravenous antibiotics" OR IV) AND

(oral OR "per oral" OR PO) AND

("oral antibiotics" OR antibiotic OR antibiotics OR macrolides OR fluoroquinolones OR

penicillins OR cephalosporins OR tetracyclines OR "anti-bacterial agents" OR

bioavailability OR bioavailable OR "oral absorption")))

AND

TS=("clinical pathway" OR "clinical pathways" OR "practice guideline" OR

"critical pathway" OR "care pathway" OR "treatment protocol" OR "clinical protocol" OR

"guideline adherence" OR guidelines OR "implementation strategy" OR "intervention study" OR

intervention OR "care bundle" OR "antibiotic stewardship" OR "antimicrobial stewardship" OR

"pharmacy-led" OR "pharmacist-led" OR "pharmacist intervention" OR "clinical pharmacist" OR

"decision support" OR "clinical decision support" OR "decision aid" OR "point-of-care intervention")

AND

TS=(hospitalized OR hospitalised OR hospitalisation OR hospitalization OR inpatient OR

inpatients OR "medical ward" OR "healthcare setting" OR "healthcare facility" OR

"secondary care" OR "tertiary care" OR "acute care" OR "surgical ward" OR

"community hospital")

AND

TS=("length of stay" OR "hospital stay" OR "duration of stay" OR "duration of therapy" OR

"treatment duration" OR "clinical outcomes" OR "outcome assessment" OR

"treatment failure" OR readmission OR "re-admission" OR "hospital discharge" OR

"early discharge" OR "discharge planning" OR "economic outcomes" OR "cost-effectiveness" OR

"cost analysis" OR "health economics" OR safety OR "clinical stability" OR

"therapeutic success")

**Scopus**

(TITLE-ABS-KEY(pneumonia OR pneumonias OR pneumoniae OR "community-acquired pneumonia" OR CAP OR "hospital-acquired pneumonia" OR HAP OR "ventilator-associated pneumonia" OR VAP OR "healthcare-associated pneumonia" OR HCAP OR "lower respiratory tract infection" OR LRTI OR "respiratory tract infection" OR RTI OR "chest infection") AND TITLE-ABS-KEY("intravenous to oral switch" OR "IV to PO switch" OR "IV to PO transition" OR "oral step-down therapy" OR "step-down therapy" OR "sequential therapy" OR "switch therapy" OR "early switch" OR "early discharge" OR IVOS OR "IV-to-oral conversion" OR "IV-PO conversion" OR ( intravenous OR "intravenous antibiotics" OR IV ) AND ( oral OR "per oral" OR PO ) AND ( "oral antibiotics" OR antibiotic OR antibiotics OR macrolides OR fluoroquinolones OR penicillins OR cephalosporins OR tetracyclines OR "anti-bacterial agents" OR bioavailability OR bioavailable OR "oral absorption" )) AND TITLE-ABS-KEY("clinical pathway" OR "clinical pathways" OR "practice guideline" OR "critical pathway" OR "care pathway" OR "treatment protocol" OR "clinical protocol" OR "guideline adherence" OR guidelines OR "implementation strategy" OR "intervention study" OR intervention OR "care bundle" OR "antibiotic stewardship" OR "antimicrobial stewardship" OR "pharmacy-led" OR "pharmacist-led" OR "pharmacist intervention" OR "clinical pharmacist" OR "decision support" OR "clinical decision support" OR "decision aid" OR "point-of-care intervention") AND TITLE-ABS-KEY(hospitalized OR hospitalised OR hospitalisation OR hospitalization OR inpatient OR inpatients OR "medical ward" OR "healthcare setting" OR "healthcare facility" OR "secondary care" OR "tertiary care" OR "acute care" OR "surgical ward" OR "community hospital") AND TITLE-ABS-KEY("length of stay" OR "hospital stay" OR "duration of stay" OR "duration of therapy" OR "treatment duration" OR "clinical outcomes" OR "outcome assessment" OR "treatment failure" OR readmission OR "re-admission" OR "hospital discharge" OR "early discharge" OR "discharge planning" OR "economic outcomes" OR "cost-effectiveness" OR "cost analysis" OR "health economics" OR safety OR "clinical stability" OR "therapeutic success"))

**PubMed/Medline**

(pneumonia OR pneumonias OR pneumoniae OR "community-acquired pneumonia" OR CAP OR "hospital-acquired pneumonia" OR HAP OR "ventilator-associated pneumonia" OR VAP OR "healthcare-associated pneumonia" OR HCAP OR "lower respiratory tract infection" OR LRTI OR "respiratory tract infection" OR RTI OR "chest infection") AND TITLE-ABS-KEY("intravenous to oral switch" OR "IV to PO switch" OR "IV to PO transition" OR "oral step-down therapy" OR "step-down therapy" OR "sequential therapy" OR "switch therapy" OR "early switch" OR "early discharge" OR IVOS OR "IV-to-oral conversion" OR "IV-PO conversion" OR ( intravenous OR "intravenous antibiotics" OR IV ) AND ( oral OR "per oral" OR PO ) AND ( "oral antibiotics" OR antibiotic OR antibiotics OR macrolides OR fluoroquinolones OR penicillins OR cephalosporins OR tetracyclines OR "anti-bacterial agents" OR bioavailability OR bioavailable OR "oral absorption" )) AND TITLE-ABS-KEY("clinical pathway" OR "clinical pathways" OR "practice guideline" OR "critical pathway" OR "care pathway" OR "treatment protocol" OR "clinical protocol" OR "guideline adherence" OR guidelines OR "implementation strategy" OR "intervention study" OR intervention OR "care bundle" OR "antibiotic stewardship" OR "antimicrobial stewardship" OR "pharmacy-led" OR "pharmacist-led" OR "pharmacist intervention" OR "clinical pharmacist" OR "decision support" OR "clinical decision support" OR "decision aid" OR "point-of-care intervention") AND TITLE-ABS-KEY(hospitalized OR hospitalised OR hospitalisation OR hospitalization OR inpatient OR inpatients OR "medical ward" OR "healthcare setting" OR "healthcare facility" OR "secondary care" OR "tertiary care" OR "acute care" OR "surgical ward" OR "community hospital") AND TITLE-ABS-KEY("length of stay" OR "hospital stay" OR "duration of stay" OR "duration of therapy" OR "treatment duration" OR "clinical outcomes" OR "outcome assessment" OR "treatment failure" OR readmission OR "re-admission" OR "hospital discharge" OR "early discharge" OR "discharge planning" OR "economic outcomes" OR "cost-effectiveness" OR "cost analysis" OR "health economics" OR safety OR "clinical stability" OR "therapeutic success"))
